# Epigenetic downregulation of STAT6 increases HIF-1α expression via mTOR/S6K/S6, leading to enhanced hypoxic viability of glioma cells

**DOI:** 10.1186/s40478-019-0798-z

**Published:** 2019-09-17

**Authors:** Soo Jung Park, Hyunmi Kim, Se Hyuk Kim, Eun-hye Joe, Ilo Jou

**Affiliations:** 1 Department of Pharmacology, School of Medicine, Ajou University, Suwon, 16499 South Korea; 2 Chronic Inflammatory Disease Research Center, Ajou University, Suwon, 16499 South Korea; 3 Department of Neurosurgery, School of Medicine, Ajou University, Suwon, 16499 South Korea

**Keywords:** STAT6, DNA hypermethylation, Glioma, HIF-1α, mTOR, Rheb

## Abstract

Multifunctional signal transducer and activator of transcription (STAT) proteins play important roles in cancer. Here, we have shown that STAT6 is epigenetically silenced in some cases of malignant glioblastoma, which facilitates cancer cell survival in a hypoxic microenvironment. This downregulation results from hypermethylation of CpG islands within the *STAT6* promoter by DNA methyltransferases. STAT6 interacts with Rheb under hypoxia and inhibits mTOR/S6K/S6 signaling, in turn, inducing increased HIF-1α translation. STAT6 silencing and consequent tumor-promoting effects are additionally observed in glioma stem-like cells (GSC). Despite recent advances in cancer treatment, survival rates have shown little improvement. This is particularly true in the case of glioma, where multimodal treatment and precision medicine is needed. Our study supports the application of epigenetic restoration of STAT6 with the aid of DNA methyltransferase inhibitors, such as 5-aza-2-deoxycytidine, for treatment of STAT6-silenced gliomas.

**Electronic supplementary material:**

The online version of this article (10.1186/s40478-019-0798-z) contains supplementary material, which is available to authorized users.

## Introduction

Epigenetic modifications comprising DNA methylations and histone modifications both qualitatively and quantitatively alter gene expression [15, 36]. Accordingly, together with genetic mutations, epigenetic modifications can produce changes in physiologic functions that contribute to various diseases, including cancers. Recent genome-wide DNA methylation analyses and large-scale epigenetic profiling studies have revealed widespread distribution of epigenetic markers as well as frequent epigenetic alterations in a variety of human malignancies, thus establishing a causative role for an altered epigenome in carcinogenesis [1]. The predominant modification of mammalian DNA is cytosine methylation, followed by adenine and guanine methylation [17]. Methylation of cytosine bases in mammalian DNA has been primarily described in the context of CpG dinucleotides. Methylation of cytosine in the promoter region may repress expression of the corresponding gene by preventing the binding of specific transcription factors or attracting mediators of chromatin remodeling, such as histone-modifying enzymes or other repressors of gene expression.

An aberrant epigenome is a major risk factor in glioblastoma (GBM), the most malignant and lethal human brain tumor [27]. Importantly, hypermethylation of the DNA repair enzyme, O^6^-methylguanine-DNA methyltransferase (MGMT), which prevents repair of aberrant methylation, is a strong prognostic biomarker for the responsiveness of GBM patients to alkylating agents, including temozolomide [49]. Furthermore, The Cancer Genome Atlas (TCGA) project recently identified a subset of GBM tumors with cancer-specific CpG island hypermethylation, referred to as a glioma CpG island methylator phenotype (G-CIMP) [34]. These CIMP-positive tumors often exhibit characteristic molecular and clinicopathologic features, suggesting that CIMP represents a distinct carcinogenic pathway. The identification of CIMP could potentially lead to a better understanding of the molecular basis of tumorigenesis as well as improved treatment of cancer patients.

Accumulating evidence supports an important role of tissue hypoxia in cancer cell growth, neovascularization, invasion, resistance to chemo/radiotherapy and, ultimately, recurrence after treatment [13]. Adaptation to hypoxia allows for cancer progression in the hypoxic environment characteristic of the tumor core. A major mechanism that mediates adaptive responses to reduced O_2_ is activation of hypoxia-inducible factor-1 (HIF-1) [41]. HIF-1 levels and activity are primarily controlled by the availability of the oxygen-sensitive HIF-1α subunits. In normoxia, prolyl hydroxylases (PHD) hydroxylate HIF-1α, triggering von Hippel–Lindau (pVHL)-mediated ubiquitination and proteasomal degradation. Conversely, hypoxia leads to inhibition of PHDs and stabilization of HIF-1α. In addition to protein stability, protein synthesis contributes to HIF-1α accumulation in response to hypoxia. In this context, several studies have shown that mTOR, a key regulator of protein synthesis, plays a significant role in HIF-1α accumulation under hypoxic conditions [2, 4, 10]. Relative to HIF-1α regulation via protein degradation, the regulatory mechanisms governing translational processes are largely unknown.

High-level expression of HIF-1α is positively correlated with tumor progression and poor prognosis in patients with GBM [44, 52]. Small molecule- or siRNA-mediated inhibition of HIF-1α can induce apoptosis in gliomas, sensitizing them to chemotherapeutic agents and impairing cancer cell migration and invasion in vitro under hypoxic conditions [6, 12]. Hypoxia also provides a niche microenvironment that maintains glioma stem-like cells (GSCs) [14, 43], a phenomenon in which HIF-1α plays a critical role [25]. Therefore, targeting HIF-1α and its associated regulators and gene targets in glioma could lead to benefits for cancer patients.

Signal transducer and activator of transcription (STAT) proteins have important roles in many biological processes [45]; thus, not surprisingly, dysregulation of STAT signaling has been reported in many human diseases, including cancers. Dysregulation of STAT signaling can result from an increase in cytokine release and receptor activation, as well as upstream kinase hyperactivity [30]. In addition to protein activity, the amount of STAT protein is important in tumorigenesis. Gain-of-function mutations in STAT3 and STAT5 have been reported in leukemia and lymphoma [20, 21, 33]. Epigenetically silenced STAT may also serve a tumor-promoting function. For example, *STAT1* and *STAT5A* genes are silenced by DNA methylation in squamous cell carcinoma of the head and neck (SCCHN) and NPM1-ALK–expressing lymphomas, respectively [51, 53]. Although aberrant STAT signaling has been linked to diverse aspects of GBM tumor progression, invasion and GSC maintenance [3, 26], the contribution of STAT gene dysregulation to tumor pathology, particularly at the epigenetic level, is unclear.

Despite recent clinical trials of targeted therapies, further advances in therapeutic strategies have stalled, possibly reflecting the complex heterogeneity of cancer cells. In this study, we demonstrated that STAT6, an important signaling molecule in adaptive immunity, is frequently silenced in gliomas where hypoxia is a prominent feature. Based on the findings, we propose that STAT6 downregulation resulting from DNA methyltransferase (DNMT)-mediated hypermethylation of promoter CpG islands facilitates accumulation of HIF-1α through mTOR activation in hypoxia and consequent enhancement of glioma cell survival. mTOR activation via STAT6 knockdown is achieved through suppression of direct interactions between STAT6 and Rheb that inhibit HIF-1α translation. Moreover, STAT6 silencing and resulting tumor-promoting effects were consistently observed in glioma stem-like cells. Recent developments in application of precision medicine to cancer treatment support the application of epigenetic restoration of STAT6 via DNA methyltransferase inhibitors (DNMTi) as a therapeutic strategy for STAT6-silenced gliomas.

## Materials and methods

### Reagents and antibodies

Cycloheximide, 5-aza-2′-deoxycytidine (5-Aza), trichostatin A (TSA), and rapamycin were purchased from Sigma-Aldrich (St. Louis, MO, USA). The indicated primary antibodies against the following proteins were used in this study: STAT1/2/3/4/5/6 (#9172/4594/12640/5097/9363/9362), DNMT1/3a/3b (#5119/3598/67259), cleaved-caspase 3/7 (#9664/9491), cleaved PARP (#5625), S6K (#2708), phospho-S6K (T389) (#9205), S6 (#2217), phospho-S6 (Ser235/236) (#2211), mTOR (#2972), 4E-BP1 (#9644), phospho-4E-BP1 (T70) (#9455), eIF4E (#2067), phospho-eIF4E (S209) (#9741), eIF4G (#2469), Rheb (#13879), TSC2 (#4308), p-TSC2 (T1462) (#3617), p-TSC2 (S1387) (#5584), ICAM1 (#4915), JAK2 (#3230), NFATC2 (#4389) and Myc taq (#2278) (Cell Signaling Technology, Danvers, MA, USA); anti-HIF-1α (610958) (BD Biosciences, San Jose, CA, USA); and anti-actin (sc-1616) and GAPDH (sc-48,167) (Santa Cruz Biotechnology, Santa Cruz, CA, USA). Secondary antibodies used were anti-goat IgG HRP (81–1620; Invitrogen, Carlsbad, CA, USA), anti-mouse IgG HRP (G-21040; Invitrogen), and anti-rabbit IgG HRP (111–035-003; Jackson Laboratories, Bar Harbor, ME, USA).

### Cell lines and tumor samples

The human glioblastoma cell lines U87MG and U373MG were obtained from the Korean Cell Line Bank (Seoul, Korea) and U251 and LN229 were kindly provided by Dr. Hee Young Kim (Seoul National University, Seoul, Republic of Korea). The cancer cell lines were routinely grown in Dulbecco’s Modified Eagle Medium (DMEM; Sigma-Aldrich) containing 10% fetal bovine serum (FBS; Gemini, West Sacramento, CA, USA) and 0.1% antibiotic-antimycotic solution (Gibco, Carlsbad, CA, USA).

Fetal normal human astrocytes (NHA) were purchased from ABM (Richmond, BC, Canada) and were cultured according to manufacturer’s direction in Prigrow X Series Medium (ABM). Glioma stem-like cells (GSCs) were established from freshly resected tumors and were cultured in neurobasal media (Gibco) supplemented with N2 (Gibco) and B27 (Invitrogen). Cultures were supplemented with 20 ng/mL of epidermal growth factor (EGF) (Invitrogen) and basic fibroblast growth factor (bFGF) (Millipore, Billerica, MA, USA) every 2–3 days.

All cells were incubated at 37 °C in a humidified atmosphere of 95% air and 5% CO_2_. For hypoxia treatment, cells were incubated in an oxygen control hypoxia chamber (Coy Laboratory Products, Grass Lake, MI, USA) at 37 °C in a humidified 5% CO_2_ environment, with the balance provided by N_2_.

Following informed consent, glioma and normal brain tissues were obtained from patients undergoing surgery at the Ajou University Hospital in accordance with Institutional Review Boards protocols. The samples were snap-frozen in liquid nitrogen and stored at − 80 °C until analysis. Detailed information of patients is provided in Additional file 2: Table S1.

### Immunoblotting

Cells were lysed in RIPA buffer (50 mM Tris-HCl, 1% NP-40, 0.25% sodium deoxycholate, 150 mM NaCl, 1 mM EDTA) supplemented with protease inhibitor cocktail and phosphatase inhibitor cocktail. The protein concentration in lysates was determined using the Bio-Rad protein assay (Bio-Rad Laboratories, Hercules, CA, USA). Proteins were separated by sodium dodecyl sulfate-polyacrylamide gel electrophoresis (SDS-PAGE) and transferred to nitrocellulose membranes. Primary antibodies were applied overnight at 4 °C. Peroxidase-conjugated secondary antibodies were applied at room temperature for 1 h, and immunoreactive protein were visualized using an enhanced chemiluminescence detection kit (WESTSAVE Gold; AbFrontier, Seoul, Korea), followed by exposure to X-ray film. Band intensities were quantified using ImageJ software (National Institutes of Health, Bethesda, MD, USA).

### Immunohistochemistry

A tissue microarray (TMA) containing glioblastoma and normal brain samples (GL806; US Biomax,Rockville, MD, USA) was used for high throughput analysis of gene expression. The TMA contains 35 cases of glioblastoma and 5 normal brain tissues, represented as duplicate cores per case. Tissue microarray slides were deparaffinized in xylene followed by rehydration. For antigen retrieval, slides were boiled in 10 mM citric acid buffer (pH 6.0) for 20 min, followed by blocking of endogenous peroxidase by incubating in 0.3% H_2_O_2_ in methanol for 30 min. Slides were blocked by incubating for 1 h in 10% normal goat serum, and then incubated overnight at 4 °C with a primary antibody against STAT6 (1:100; Abcam, Cambridge, MA, USA) and then with biotinylated goat anti-rabbit IgG secondary antibody (1:400) for 1 h. The antigen-antibody reaction was visualized using the Vector SG peroxidase substrate in a Vectastain Elite ABC kit (Vector Laboratories, Burlingame, CA, USA). Slides were counterstained with Harris hematoxylin solution (Sigma), subjected to a dehydration process, then briefly washed in xylene and mounted with VectaMount Permanent Mounting Medium (Vector Laboratories). For the quantitative analysis, a Histo score (H score) was calculated based on the staining intensity and percentage of stained cells. The intensity score was defined as follows: 1, weak staining; 2, intermediate staining; 3, strong staining. The H score was determined by the formula: 3 × percentage of strong staining + 2 × percentage of moderate staining + percentage of weak staining, giving a range of 0 to 300 for the H scores.

### Reverse transcription and quantitative real-time PCR analysis

Total RNA was isolated using RNAiso Plus (TaKaRa, Otsu, Japan), and cDNA was synthesized using avian myeloblastosis virus reverse transcriptase (New England Biolabs) and oligo (dT) primers (Promega, Madison, WI, USA), according to the manufacturer’s instructions. For qPCR, amplification reactions were performed using a Thermal Cycler Dice Real-Time System (TaKaRa) with SYBR Premix Ex Taq master mix (TaKaRa) according to the manufacturer’s instructions. The primers used for RT-qPCR (Bioneer, Daejeon, Korea) are described in Additional file 2: Table S2.

### Methylation-specific PCR and pyrosequencing of bisulfate-converted genomic DNA

The methylation status of *STAT6* promoter CpG islands was analyzed using methylation-specific PCR (MSP) and pyrosequencing. Genomic DNA (gDNA) was extracted from human glioma and normal brain tissue and U373MG and U87MG cell lines using DNeasy Blood & Tissue kits (Qiagen Hilden, Germany). Bisulfite conversion of gDNA was performed using the EpiTect Bisulfite Kit (Qiagen) and then the converted DNA was amplified with primers specific to methylated or unmethylated DNA using an EpiTect MSP Kit (Qiagen), according to the manufacturer’s instructions. The methylation-specific primer sequences in *STAT6* promoter CpG islands were 5′-GCG TCG AGT TAA TTT TTT TC-3′ (forward) and 5′-CGC TTA ATA ACC TAA ACT CGC-3′ (reverse); the unmethylation-specific primer sequences were 5′-GGT GTT GAG TTA ATT TTT TTT-3′ (forward) and 5′-CCA CTT AAT AAC CTA AAC TCA C-3′ (reverse). After the reaction was complete, products were analyzed by electrophoresis on a 2.5% agarose gel.

Pyrosequencing was performed by Genomictree (Daejeon, South Korea) using standard protocols. Briefly, bisulfite-modified gDNA was prepared using an EZ DNA Methylation-Lighting kit (Zymo Research, USA), and then converted gDNA was amplified by PCR. The reaction conditions were as follows: denaturation at 95 °C for 10 min, followed by 45 cycles of 95 °C for 30 s, 58 °C for 30 s, 72 °C for 30 s, and a final extension at 72 °C for 5 min. Pyrosequencing was performed on a PyroMark ID system using a Pyro Gold reagents kit (Qiagen) according to the manufacturer’s instruction without further optimization. The methylation percentage was calculated based on the average degree of methylation at 2–5 CpG sites formulated in pyrosequencing. Each primer was designed using Pyrosequencing Assay Design Software v2.0 (Qiagen). Primer sequences are listed in Additional file 2: Table S3.

### Knockdown and overexpression experiments

For knockdown of specific proteins, cells were transfected for 72 h with siRNA (final RNA concentration, 25 nM) using DharmaFECT 2 reagents (Dharmacon), according to the manufacturer’s instructions. SMART pool siRNA against STAT6 (L-006690-00) (Dharmacon Thermo Scientific) and non-targeting control siRNAs (D-001810-10) were purchased from Dharmacon (Lafayette, CO, USA); siRNA against DNMT1 (1786–1), DNMT3b (1789–1), HIF-1α (3091–1) and a corresponding negative control (SN-1002) were purchased from Bioneer (Korea). siRNA against DNMT3a was chemically synthesized by Bioneer (Korea). The siRNA sequences are as follows: 5′-CAG GAG AUG AUG UCC AAC CC-3′ (sense) and 5′-GGG UUG GAC AUC AUC UCC UG-3′ (antisense).

STAT6 was overexpressed by transiently transfecting U373MG cells with pCMV6-Myc-DDK-STAT6 (RC210065; Origene Technologies) or mock control vector (PS100001; Origene Technologies) using TurboFectin 8.0 (OriGene Technologies, Inc.) according to the manufacturer’s recommendations. All assays were performed at least 48 h after transfection. STAT6 deletion mutants were generated from human STAT6 cDNA (RC210065, Origene) by PCR amplification using the appropriate primers. STAT6 deletion mutants (Δ1–260 (d1), Δ261–430 (d2), Δ431–522(d3), and Δ523–622 (d4)) were cloned into the pCMV6-entry vector (PS100001, Origene). Primer sequences are listed in Additional file 2: Table S4.

### Immunofluorescence and confocal microscopy

U373MG cells cultured on poly-d-lysine–coated coverslips were fixed with 4% (v/v) paraformaldehyde at 4 °C for 15 min and permeabilized by incubating for 5 min in phosphate-buffered saline (PBS) containing 0.1% Triton X-100. Fixed cells were incubated with anti-STAT6 (611,290, BD Biosciences) and HIF-1α (ab51608, Abcam) antibodies at 4 °C overnight, followed by incubation with Alexa Flour 488- or Alexa Flour 543-conjugated secondary antibodies (Molecular Probes, Eugene, OR, USA) for 2 h. Cells were mounted with DAPI-containing mounting solution and observed under a confocal microscope (LSM510; Carl Zeiss, Jena, Germany).

### Nascent HIF-1α synthesis assay

Newly synthesized proteins were detected using the Click-IT method (Life Technologies), according to the manufacturer’s instructions. Briefly MOCK- or full-length STAT6-transfected U373MG cells or control siRNA- or STAT6 siRNA-transfected U87MG cells were exposed to hypoxia for 18 h. Cells were subsequently washed with PBS, incubated in methionine-free DMEM for 1 h, then pulsed with 50 μM of the methionine analog, L-azidohomoalanine (AHA) (Cat # C10102; Life Technologies), for 2 h, after which cells were harvested. AHA-incorporated proteins were labeled with biotin using a Click-iT Biotin Protein Analysis Detection Kit (Cat# C33372; Invitrogen). Biotin-labeled proteins were pulled down using a streptavidin-agarose bead slurry (Pierce, Rockford, IL, USA) and analyzed by immunoblotting using an anti-HIF-1α antibody.

### Immunoprecipitation

Cells were lysed in CHAPS buffer (40 mM HEPES pH 7.4, 120 mM NaCl, 1 mM EDTA, 0.3% CHAPS) supplemented with protease inhibitor cocktail and phosphatase inhibitor cocktail. Lysates (300 μg) were incubated with antibody (1 μg of anti-mTOR, STAT6, eIF4E or isotype control antibodies) at 4 °C overnight. Magnetic protein G Dynabeads (Invitrogen) were then added, and tubes were rotated for an additional 2 h. After washing five times with lysis buffer, proteins were eluted from beads with 2X Laemmli buffer at 95 °C for 10 min, followed by resolution by SDS-PAGE.

### Cell viability assay

Cell viability was assessed using the EZ-Cytox Cell Viability Assay kit (Daeil Lab Service, Seoul, Korea). Control siRNA- or STAT6 siRNA-transfected U87MG cells were exposed to hypoxia for 5 days. The Ez-Cytox Kit reagent was then added to the medium and cells were incubated at 37 °C for 2 h. The plate was read on an iMark microplate absorbance reader (Bio-Rad) at a wavelength of 450 nm.

Cell viability was also assessed using the LIVE/DEAD™ Viability/Cytotoxicity Kit (Molecular Probes). Control siRNA- or STAT6 siRNA-transfected U87MG cells exposed to hypoxia for 5 days were washed twice with PBS and then incubated in presence of 0.5 μL/mL Calcein-AM (Live/green) and 2 *μ*L/mL EthD-1(Dead/green) for 20 min at room temperature in dark. Fluorescence images were collected using a fluorescence microscope (Zeiss Axiovert 200 M; Carl Zeiss).

### Flow cytometry

U373MG cells were stained with propidium iodide (PI) (Sigma-Aldrich) for cell cycle analysis, or with PI and annexin V-FITC (BD Biosciences) for detection of apoptosis. The distribution of cells in the different phases of the cell cycle (based on differences in DNA content) and apoptosis-positive cells were determined by flow cytometry using a FACSCalibur flow cytometer (BD Biosciences).

#### cDNA microarray

U373MG cells were transfected with control siRNA or STAT6 siRNA for 2 days. One day after transfection, cells were incubated with or without 5-Aza (300 nM) for 2 days and then the medium was replaced with fresh medium (without drug). Total RNA was extracted from cells on day 6 after drug withdrawal using the TRIzol reagent (Invitrogen). RNA samples were converted to labeled single-stranded DNA and hybridized to the GeneChip Human 2.0 ST array (Affymetrix). After hybridization, the chips were stained and washed in a Genechip Fluidics Station 450 and scanned on a GCS3000 Scanner (Affymetrix). Array data export, processing, and analysis were performed using Affymetrix GeneChip Command Console software. Raw data were extracted automatically in the Affymetrix data extraction protocol using the provided Affymetrix GeneChip Command Console Software (AGCC). After importing CEL files, the data were summarized and normalized using the robust multi-average (RMA) method implemented in Affymetrix Expression Console Software (EC). The results of gene-level RMA analysis were exported, and differentially expressed genes (DEGs) were analyzed by performing a comparative analysis of fold-change between test sample and control sample. A hierarchical cluster analysis was performed on the DEG set using complete linkage and Euclidean distance as a measure of similarity. Gene-Enrichment and Functional Annotation analyses of the significant probe list were performed using Gene Ontology (http://geneontology.org/). The R statistical language v. 3.1.2. (www.r-project.org) was used for all statistical tests and visualization of differentially expressed genes.

#### ELISA

Control siRNA- or STAT6 siRNA-transfected U373MG cells were treated with or without 5-Aza, after which the concentrations of CCL2, CCL20, IL-8, and IL-1 in culture supernatants were detected using commercial ELISA kits, according to the manufacturer’s instructions. Optical density was determined on an iMark microplate absorbance reader (Bio-Rad) at a wavelength of 450 nm. Human CCL2 (DCP00), CCL20/MIP-3α (DM3A00), IL-8/CXCL8 (D8000C), and IL-1 β/IL-1F2 (DLB50) ELISA Kit were purchased from R&D Systems (Minneapolis, MN, USA).

### Statistical analysis

GraphPad Prism 5.0 was used for statistical analyses. Differences between two groups were analyzed using an unpaired two-tailed Student’s t-test. All results are presented as means and standard deviation (S.D.) of at least three independent experiments. The results were considered statistically significant at *p*-values < 0.05.

## Results

### STAT6 expression is downregulated in human glioma

**Fig. 1:**
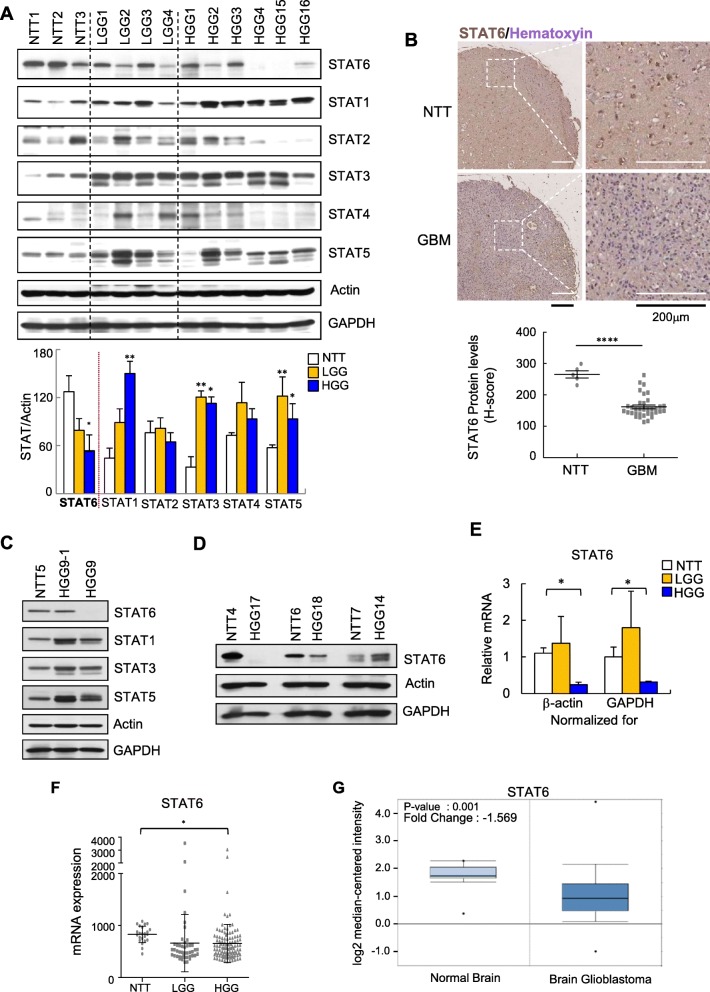
STAT6 is downregulated in glioma. **a** Immunoblot of the indicated STATs in non-tumor brain tissue (NTT), low-grade glioma tissue (LGG), and high-grade glioma tissue (HGG) (top) and summary data (bottom) showing actin-normalized levels of STATs. **b** Representative immunohistochemical staining of STAT6 in normal and GBM tissue (top) and summary data (bottom) showing the quantification (H-score, see Materials and Methods) of STAT6 staining. **c** Immunoblot of the indicated STATs in paired normal (NTT5), primary (HGG9–1) and recurrent GBM (HGG9) samples. N, normal; T, tumor. **d** Immunoblot of STAT6 in GBM (HGG17 and HGG18) and anaplastic oligodendroglioma (HGG14) and paired normal tissues from each. NTT4, HGG17-paired; NTT6, HGG18-paired; NTT7, HGG14-paired. **e** STAT6 mRNA levels in NTT (*n* = 3), LGG (*n* = 3) and HGG (*n* = 3), determined by RT-qPCR. Transcript levels were normalized to GAPDH or actin levels. **f** Expression of STAT6 from GEO data set GDS1962 containing 23 non-tumor (NTT), 45 grade II (LGG), and 31 and 81 grade III and IV (HGG) samples (*p < 0.05, **p < 0.005, and ***p < 0.0005 vs. NTT). **g** Expression of STAT6 from Oncomine data set TCGA brain consisting of normal brain (*n* = 10) and brain glioblastoma (*n* = 542) samples

To explore the function and significance of STAT proteins in gliomas, we first examined the expression levels of individual STAT proteins in human glioma specimens. Western blot analyses demonstrated that, whereas STAT2 and STAT4 showed no prominent or consistent changes in expression, STAT1, STAT3 and STAT5 expression were increased in glioma tissues compared with neighboring non-tumor tissue. In contrast, STAT6 expression, which was apparent in non-tumor tissue, was decreased in glioma tissues, exhibiting an inverse relationship with tumor grade (Fig. 1a). Although it is well known that STAT3 is upregulated in glioma and plays a role in tumor progression and survival, little is known about STAT6 in this context. To obtain further evidence of STAT6 downregulation in gliomas, we performed immunohistochemical analyses using a brain glioma tissue array (see Materials and Methods). Compared to non-tumor tissue, GBM tissue exhibited a significantly lower percentage of positive staining for STAT6 (Fig. 1b). Interestingly, in a primary GBM specimen in which STAT6 downregulation was not observed, STAT6 expression was clearly reduced following recurrence of glioma (Fig. 1c). STAT6 was also downregulated in astrocytoma, but not in oligodendroglioma (Fig. 1d). A transcript analysis revealed that this downregulation of STAT6 protein expression resulted from decreased mRNA production (Fig. 1e). In line with our results, a published microarray-based, high-throughput assessment (GEO DataSet record GDS1962) showed that STAT6 mRNA is reduced in glioma tissues compared with non-tumor tissue (Fig. 1f). Decreased STAT6 mRNA expression in GBM was also shown in TCGA brain in Oncomine database (Fig. 1g). Taken together, these observations demonstrate that STAT6 transcripts are downregulated in glioma tissues, leading to reduced STAT6 protein expression.

### DNMT1-dependent DNA methylation inhibits STAT6 expression

**Fig. 2:**
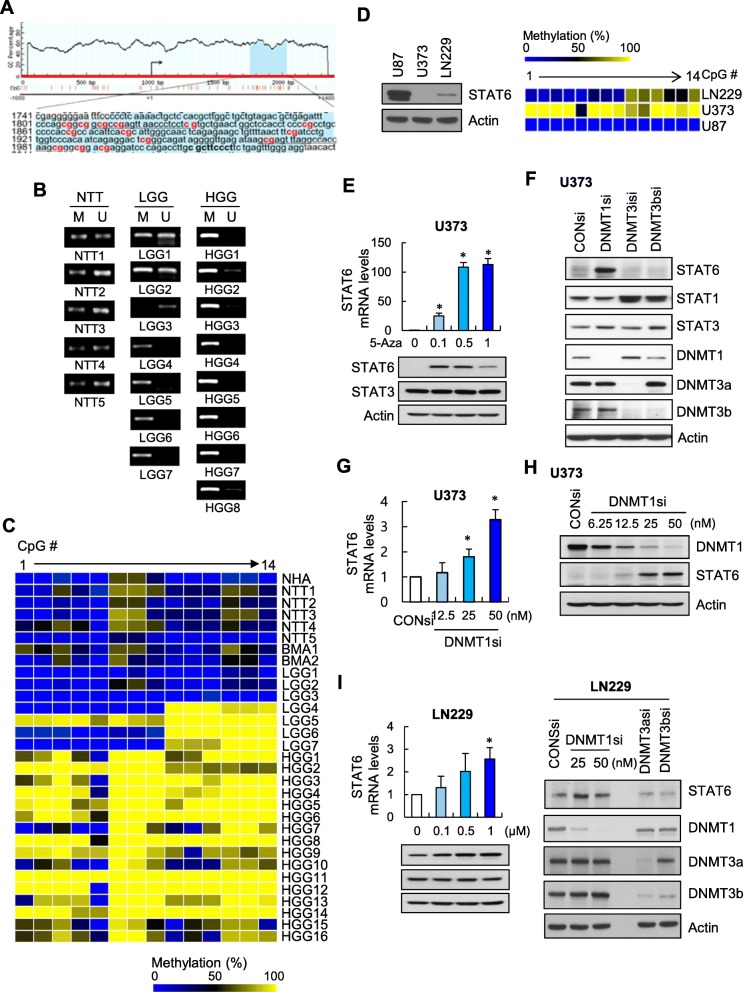
DNMT1-dependent DNA methylation inhibits STAT6 expression. **a** Distribution of CpG islands in the − 1000 to + 1400 *STAT6* promoter region. Two underlined sequences show the locations of MSP-PCR primers; 14 analyzed CpG sites are shown in bold red font. **b** MSP of *STAT6* promoter CpG islands in non-tumor brain tissue (NTT), low-grade glioma tissue (LGG), and high-grade glioma tissue (HGG). M, methylated product; U, unmethylated product. **c** Bisulfite sequencing of 14 CpG sites in *STAT6* promoter CpG islands in normal human astrocytes (NHAs), NTT, brain metastatic adenocarcinoma (BMA), LGG, and HGG tissue. Each square represents 1 CpG analyzed, and colors indicate the percentage of methylation. **d** Immunoblot of STAT6 (left) and bisulfite sequencing (right) of *STAT6* promoter CpG islands in the indicated GBM cell lines. **e** RT-qPCR of STAT6 (top) and immunoblot of indicated proteins (bottom) in U373MG cells treated with indicated doses of 5-Aza for 72 h. Results are presented as means ± SD (error bars) of three independent experiments (*p < 0.05 and **p < 0.01 vs. untreated cells). **f** Immunoblot of the indicated STATs and DNMTs in U373MG cells transfected with the indicated DNMT siRNA (DNMTsi) or control siRNA (CONsi). **g**, **h** U373MG cells transfected with CONsi or indicated doses of DNMT1si, followed by quantification of STAT6 mRNA by RT-qPCR (**g**) and immunoblotting for DNMT1 and STAT6 in cells from g (**h**). Results are presented as means ± SD (error bars) of three independent experiments (*p < 0.05 vs. CONsi-transfected cells). **i** RT-qPCR of STAT6 in LN229 cells treated with indicated doses of 5-Aza for 72 h (left) and immunoblot of STAT6 and the indicated DNMTs in LN229 cells transfected with the indicated DNMT siRNA or control siRNA (right). Results are presented as means ± SD (error bars) of three independent experiments (*p < 0.05 and **p < 0.01 vs. untreated cells)

To establish the mechanisms underlying downregulation of STAT6 transcript levels in human glioma, we first investigated the possibility of epigenetic silencing via CpG island hypermethylation. One CpG island upstream of the transcription start site of *STAT6* gene (+ 756 to + 1033 bp), containing 14 CpG sites, was predicted using MethPrimer (http://www.urogene.org/methprimer/) (Fig. 2a). An examination of methylation status in non-tumor tissue and glioma tissue using methylation-specific PCR (MSP) revealed that CpG islands in the *STAT6* promoter were more frequently methylated in high-grade gliomas than low grade gliomas, while relatively weak methylation was found in non-tumor tissue (Fig. 2b). To validate these findings, we analyzed 14 CpG sites located at the core of the CpG islands in non-tumor tissue and glioma tissue using bisulfite sequencing, which has increased sensitivity compared with MSP. In line with the results of MSP analyses, samples from high-grade gliomas showed prominent methylation (Fig. 2c), demonstrating a strong correlation between STAT6 downregulation and methylation of the *STAT6* promoter in glioma.

Next, we explored whether promoter methylation directly mediates the silencing of STAT6 in glioma tissue. First, we examined whether STAT6 expression was increased by treatment with 5-aza-2-deoxycytidine (5-Aza), a DNMT inhibitor. In the GBM cell lines, U373MG and LN229, STAT6 expression was barely detectable and methylation was more frequent compared with U87MG cells (Fig. 2d). In U373MG cells, 5-Aza induced a concentration-dependent increase in STAT6 mRNA and protein (Fig. 2e). However, the histone deacetylase inhibitor trichostatin A (TSA), had little effect on STAT6 expression (Additional file 1: Figure S1), suggesting that methylation and not acetylation of DNA plays a dominant role in STAT6 silencing in glioma.

To further confirm the involvement of DNMT, we examined STAT6 expression level following small interfering RNA (siRNA)-mediated knockdown of the major mammalian DNMTs, DNMT1, DNMT3a and DNMT3b. In U373MG cells, STAT6 expression was restored by silencing DNMT1 with DNMT1si, but not by silencing either DNMT3a or DNMT3b (Fig. 2f). We further found that the extent to which STAT6 expression increased in U373MG cells was dependent on the concentration of DNMT1si (Fig. 2g and h). Similar effects were observed in experiments using LN229 cells (Fig. 2i). Taken together, these results suggest a role for DNMT1 in STAT6 silencing in glioma.

### STAT6 expression regulates HIF-1α protein synthesis

**Fig. 3:**
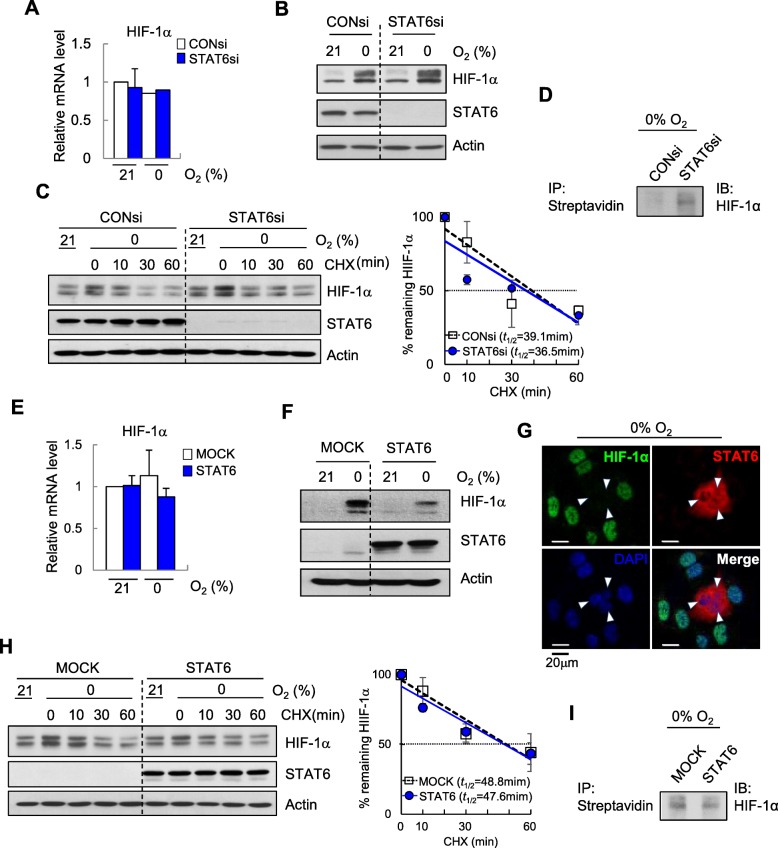
STAT6 negatively regulates HIF-1α protein synthesis. **a**, **b** U87MG cells transfected with non-targeting control siRNA (CONsi) or pooled siRNA targeting STAT6 (STAT6si) were exposed to 0% O_2_ for 18 h, followed by quantification of HIF-1α mRNA by RT-qPCR (**a**) and immunoblotting for HIF-1α and STAT6 (**b**). **c** Immunoblot of HIF-1α in CONsi or STAT6si-transfected U87MG cells exposed to 0% O_2_ for 18 h_,_ followed by treatment with or without CHX (25 μM) for the indicated times (left) and summary data (right) showing actin-normalized HIF-1α levels, expressed as a percentage relative to time 0 (as 100%). Results are presented as means ± SD (error bars) of three independent experiments. **d** Immunoblot of newly synthesized HIF-1α in AHA-labeled U87MG cells exposed to 18 h of 0% O_2_. **e, f** U373MG cells transfected with empty vector (MOCK) or Myc-tagged full-length STAT6 (STAT6) were exposed to 0% O_2_ for 18 h, followed by quantification of HIF-1α mRNA by RT-qPCR (**e**) and immunoblotting for HIF-1α and STAT6 (**f**). **g** IF staining of HIF-1α in STAT6-transfected U373MG cells exposed to 0% O_2_ for 18 h. **h** Immunoblot of HIF-1α in MOCK- or STAT6-transfected U373MG cells treated as in **c** (left) and summary data (right) showing actin-normalized HIF-1α levels, expressed as a percentage relative to time 0 (as 100%). Results are presented as means ± SD (error bars) of three independent experiments. **i** Immunoblot of newly synthesized HIF-1α in AHA-labeled U373MG cells exposed to 18 h of 0% O_2_

Next, we explored the functional relevance of STAT6 downregulation in GBM cells. Given the characteristic hypoxic microenvironment of glioma, we initially assessed whether STAT6 is related to HIF-1α expression, since HIF-1α is the critical mediator to hypoxic adaptation and survival. Experiments were performed using U87MG cells with high STAT6 expression and low methylation levels. Interestingly, while siRNA-mediated knockdown of STAT6 did not induce significant differences in HIF-1α mRNA levels under hypoxia (Fig. 3a), HIF-1α protein levels were clearly increased (Fig. 3b).

We then tested whether STAT6 affects HIF-1α accumulation through regulation of its protein degradation. In the presence of cycloheximide (CHX) to block de novo protein synthesis, the half-life of HIF-1α was similar in control and STAT6 knockdown cells (Fig. 3c), indicating that STAT6 is not involved in HIF-1α protein degradation. An assay kit that detects only newly synthesized protein confirmed that de novo synthesis of HIF-1α was increased in the absence of STAT6 (Fig. 3d).

To validate this finding, we performed STAT6 gain-of-function experiments in U373MG cells with barely detectable STAT6 and high promoter methylation levels. While HIF-1α mRNA levels showed no differences under hypoxic conditions (Fig. 3e), HIF-1α protein expression was increased in mock-transfected cells under hypoxia and conversely reduced in STAT6-overexpressing cells (Fig. 3f). This result was supported by immunofluorescence (IF) staining showing decreased HIF-1α intensity in STAT6-expressing cells (Fig. 3g). Assessment of protein stability using cycloheximide revealed a similar half-life of HIF-1α in control and STAT6-overexpressing cells (Fig. 3h), further confirming the non-involvement of STAT6 in HIF-1α protein degradation. These findings were supported using an assay kit that specifically detects newly synthesized protein (Fig. 3i).

### Regulation of HIF-1α protein synthesis by STAT6 is mediated via mTOR signaling

**Fig. 4:**
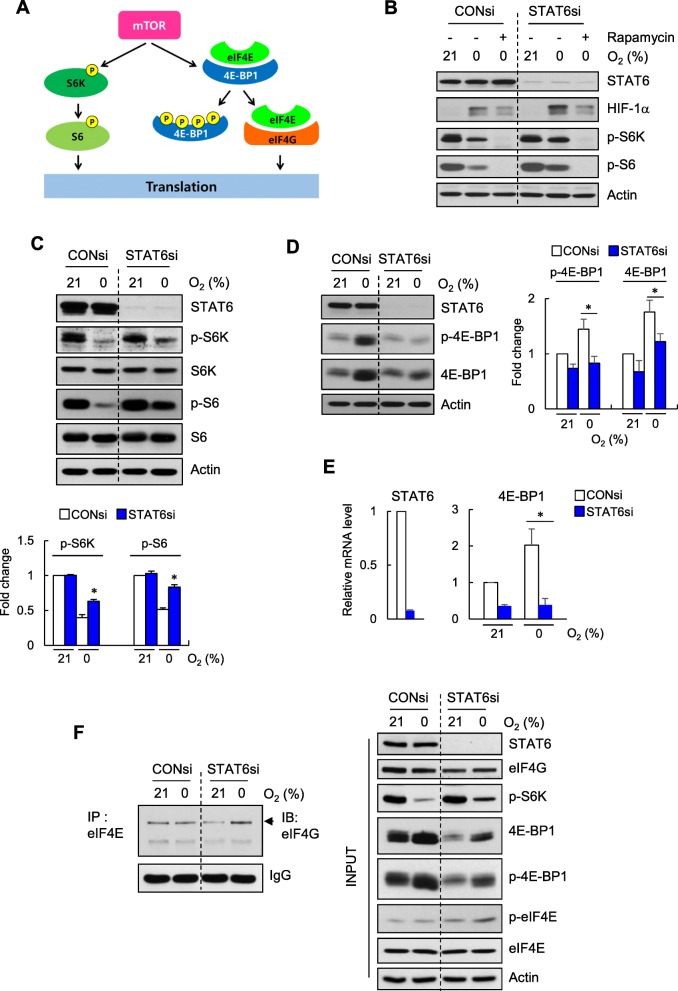
STAT6 negatively regulates mTOR signaling. **a** Schematic of downstream mTOR signaling. **b** Immunoblot of HIF-1α, p-S6K, p-S6 and STAT6 in CONsi or STAT6si -transfected U87MG cells exposed to 0% O_2_ for 18 h, with or without rapamycin (1 nM). **c** Immunoblot of the indicated proteins (top) and summary data (bottom) showing actin-normalized p-S6K and p-S6 levels expressed as fold-changes relative to that in CONsi-transfected cells exposed to 21% O_2_ in siRNA-transfected U87MG cells exposed to 0% O_2_ for 18 h. Results are presented as means ± SD (error bars) of three independent experiments (*p < 0.05). **d** Immunoblot of the indicated proteins (left) and summary data (right) showing actin-normalized p-4E-BP1 and 4E-BP1 levels expressed as fold-changes relative to that in CONsi-transfected cells exposed to 21% O_2_ in siRNA-transfected U87MG cells exposed to 0% O_2_ for 18 h. Results are presented as means ± SD (error bars) of three independent experiments (*p < 0.05). **e** RT-qPCR of 4E-BP1 in siRNA-transfected U87MG cells exposed to 0% O_2_ for 18 h. Results are presented as means ± SD (error bars) of three independent experiments (*p < 0.05). **f** Immunoblot of eIF4G in eIF4E IPs from U87MG cells transfected with CONsi or STAT6si and exposed to 0% O_2_ for 10 h

Mammalian target of rapamycin (mTOR) regulates numerous components of protein synthesis, including ribosomal proteins and initiation and elongation factors (Fig. 4a). Since our data suggest that STAT6 inhibits HIF-1α at the translational level, we assessed the involvement of mTOR signaling using rapamycin, a chemical inhibitor of mTOR. Rapamycin effectively suppressed hypoxia-induced HIF-1α in both control and STAT6 knockdown cells (Fig. 4b). In view of the finding that mTOR pathways comprise S6K-S6 and 4E-BP1 (Fig. 4a), we further examined the activation status of downstream effectors of mTOR. Phosphorylation levels of both S6K and its target S6, which were decreased in a hypoxic microenvironment, were partially restored in the absence of STAT6 while the total protein levels of S6K and S6 showed no changes (Fig. 4c). In the case of 4E-BP1, another key target of mTOR, both phosphorylated and total protein levels were increased in hypoxia, which were suppressed in the absence of STAT6 (Fig. 4d). The corresponding increase in 4E-BP1 mRNA expression in hypoxia and its suppression by STAT6si (Fig. 4e) indicated that phosphorylation and protein levels reflect transcriptional patterns. In STAT6 knockdown cells, restoration of the suppressed mTOR signaling in hypoxia facilitated eIF4E binding to eIF4G (Fig. 4f).

In line with STAT6 knockdown experiments, hypoxia-induced suppression of p-S6K-S6K signaling was further decreased in STAT6-overexpressing U373MG cells (Additional file 1: Figure S2A). However, protein and transcript levels of 4E-BP1 and p-4E-BP1 displayed no significant changes following STAT6 overexpression (Additional file 1: Figure S2B and C).

### STAT6 interacts directly with Rheb, leading to mTOR inhibition

**Fig. 5:**
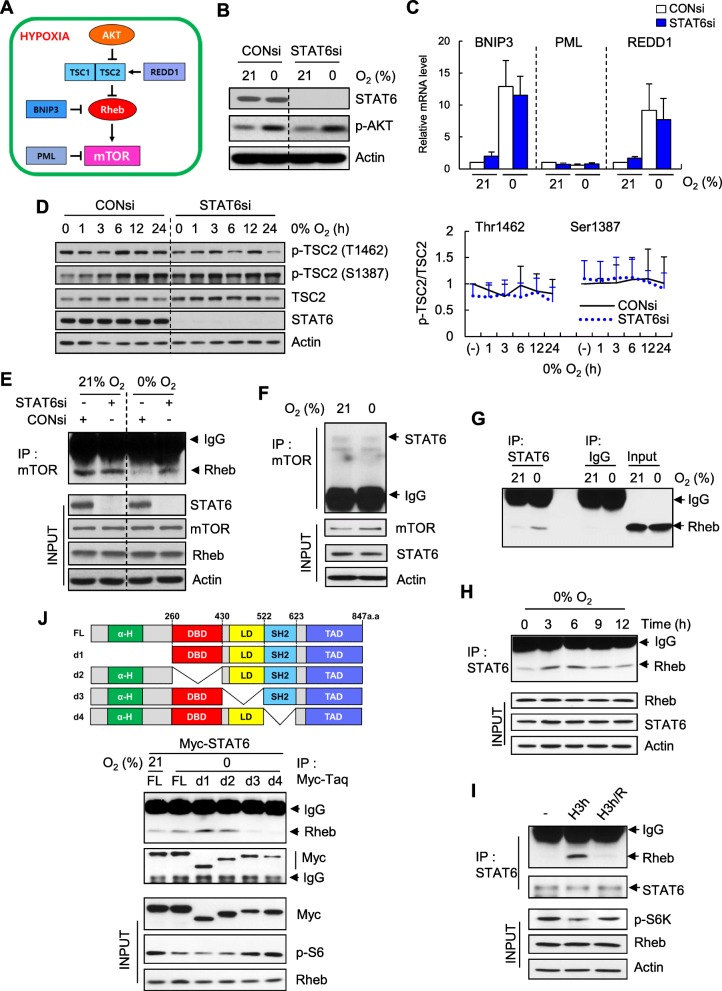
STAT6 interacts with Rheb and inhibits mTOR signaling under hypoxic conditions. **a** Schematic of inhibition of mTOR signaling under hypoxic conditions. **b C** U87MG cells transfected with non-targeting control siRNA (CONsi) or pooled siRNA targeting STAT6 (STAT6si) were exposed to 0% O_2_ for 18 h, followed by immunoblotting for STAT6 and p-AKT (**b**), and quantification of BNIP3, PML, and REDD1 mRNA by RT-qPCR (**c**). **d** Immunoblot of p-TSC2, TSC2, and STAT6 in cells exposed to 0% O_2_ for the indicated periods (left) and summary data (right) showing p-TSC2 levels, expressed as fold changes relative to that in CONsi-transfected cells exposed to 21% O_2_. **e** Immunoblot of Rheb in mTOR immunoprecipitates (IPs) from U87MG cells transfected with CONsi or STAT6si and exposed to 21% or 0% O_2_ for 10 h. **f** Immunoblot of STAT6 in mTOR IPs from U87MG cells exposed to 0% O_2_ for 6 h. **g** Immunoblot of Rheb in STAT6 IPs from U87MG cells exposed to 0% O_2_ for 10 h. IgG IPs were used as a negative control. **h** Immunoblot of Rheb in STAT6 IPs from U87MG cells exposed to 0% O_2_ for the indicated periods. **i** Immunoblot of Rheb in STAT6 IPs from U87MG cells exposed 0% O_2_ for 3 h (H3h), followed by reoxygenation for 12 h (H3h/R). **j** Top, schematic of STAT6 deletion mutants. α-H, a-helicase domain; DBD, DNA-binding domain; LD, linker domain; SH2: src homology 2 domain; TAD, transactivation domain. FL, full length; d1-d4, deletion of residues 1–260 (d1), 261–430 (d2), 431–522(d3), or 523–622 (d4), respectively. Bottom, immunoblot of Rheb in Myc-tagged STAT6 (Myc-STAT6) IPs from U373MG cells transfected with the indicated Myc-STAT6 deletions and exposed to 0% O_2_ for 6 h

The mechanism underlying STAT6-mediated inhibition of mTOR activity under hypoxia was further investigated. The TSC2-Rheb signaling axis induced suppression of mTOR activity under hypoxia (Fig. 5a). However, STAT6 did not affect activity of the upstream kinase AKT (Fig. 5b). No significant differences in mRNA induction of genes regulating DNA damage and development, including REDD 1, BNIP3 and PML (Fig. 5c), or TSC2 phosphorylation at T1462 or S138 (Fig. 5d) were evident between control-, and STAT6 siRNA-transfected U87MG in hypoxic conditions.

Accordingly, we considered the possibility that STAT6 interferes with Rheb-mTOR interactions, thereby suppressing mTOR activity. Interestingly, hypoxia-induced Rheb-mTOR dissociation was maintained in STAT6 knockdown cells (Fig. 5e). To further examine the effects of STAT6 on Rheb-mTOR binding, we analyzed the interactions of STAT6 with Rheb or mTOR. STAT6 interacted with Rheb (Fig. 5g) but not mTOR (Fig. 5f), with a peak representing optimal binding at 3–6 h after hypoxia (Fig. 5h). Moreover, under reoxygenation conditions, hypoxia-induced STAT6 binding to Rheb was suppressed (Fig. 5i), indicating the necessity of hypoxic conditions for STAT6-Rheb interactions. For further analysis of STAT6-Rheb interactions, we mapped the Rheb-binding region of STAT6 using deletion mutants of each domain. Deletion of either the Linker Domain (LD) or SH2 domain (SH) abolished STAT6 binding to Rheb under hypoxic conditions (Fig. 5j). Upon inhibition of STAT6-Rheb interactions (via transfection of d3 or d4), S6 phosphorylation was restored, even in hypoxic conditions (Fig. 5j, INPUT), signifying inhibitory effects of STAT6 on mTOR signaling through interactions with Rheb.

### STAT6 expression promotes glioma cell death

**Fig. 6:**
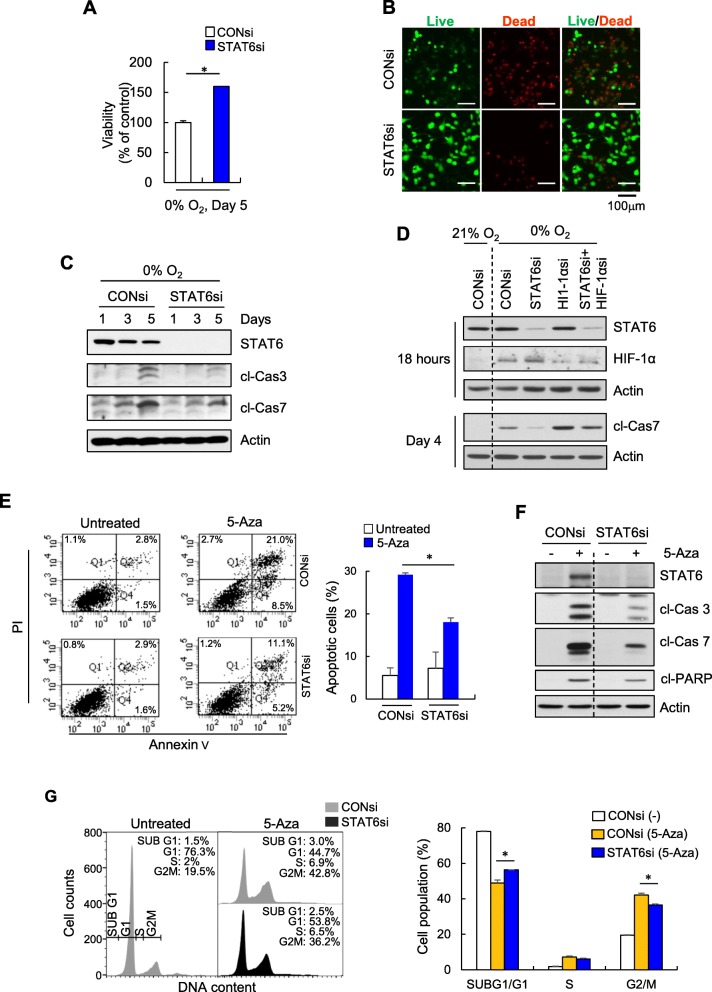
STAT6 regulates glioma cell viability. **a**, **b** U87MG cells transfected with CONsi or STAT6si were exposed to 0% O_2_ for 5 days, and then assessed by WST viability assay (**a**) and LIVE (green)/DEAD (red) assay (**b**). Results are presented as means ± SD (error bars) of three independent experiments (*p < 0.05 vs. CONsi-transfected cells). **c** Immunoblot of STAT6, cleaved (cl) caspase 3 (Cas 3), and cl-Cas 7 in siRNA-transfected U87MG cells exposed to 0% O_2_ for indicated days. **d** Immunoblot of STAT6, HIF-1α, and cl-Cas 7 in indicated siRNA-transfected U87MG cells exposed to 0% O_2_ for indicated times. **e, f** Two days after transfecting with CONsi or STAT6si, U373MG cells were incubated with or without 5-Aza (300 nM) for 2 days, and incubated in fresh medium (without drugs) for 6 days. Flow cytometry dot plots of annexin V and propidium iodide (PI) staining and summary data showing the percentage of annexin V-positive cells (**e**) and immunoblot of STAT6, cl-Cas-3 and -7, and cl- PARP (**f**). Results are presented as means ± SD (error bars) of three independent experiments (*p < 0.05). **g** Flow cytometry histogram plots of PI-stained DNA content (left) and summary data (right) showing the percentage of cells in each cell cycle phase. Results are presented as means ± SD (error bars) of three independent experiments (*p < 0.05)

To address the functional significance of STAT6-regulated HIF-1α expression in GBM cells, we initially examined the impact of STAT6 on cell viability under hypoxic microenvironments. In U87MG cells, siRNA-mediated knockdown of STAT6 led to increased cell viability (Fig. 6a and b) and decreased expression of apoptosis markers (Fig. 6c). Next, we examined the interrelationships among STAT6, HIF-1α and apoptosis by examining the expression patterns of HIF-1α and apoptosis markers at 18 h and 4 days after hypoxia, respectively. Hypoxia-induced cell death was decreased in the presence of STAT6si, which was reversed in double knockdown cells. In parallel, hypoxia-induced cell death was potentiated by HIF-1αsi and, conversely, attenuated in double knockdown cells, indicating that HIF-1α expression is dependent on STAT6 (Fig. 6d).

In STAT6 gain-of function experiments, STAT6-overexpressing U373MG cells exhibited decreased viability (Additional file 1: Figure S3A) along with increased expression of apoptosis markers (Additional file 1: Figure S3B and C).

In recent years, hypomethylating agents, such as 5-azacitidine and decitabine (5-Aza), have been clinically applied for treatment of myelodysplasia. Interestingly, these agents exert DNA methyltransferase (DNMT) inhibition effects at significantly lower concentrations than those required for cancer cell killing effects. Moreover, effects are sustained even after washout of drugs. In our cell culture model, the apoptotic cell content was significantly induced 3 days after washout of 5-Aza at concentrations ≥100 nM, which continued to increase until 6 days (Additional file 1: Figure S4).

To test whether epigenetic restoration of STAT6 mediates apoptotic effects of 5-Aza, STAT6-silenced U373MG cells were treated with 5-Aza. Transient 5-Aza treatment increased apoptotic cell death to 29 ± 0.5%, which was decreased to 18 ± 2.5% in the presence of STAT6si (Fig. 6e). Effects of STAT6 knockdown on apoptosis were further confirmed by reduced cleavage of capase-3, caspase-7 and poly-(ADP-ribose) polymerase (PARP) (Fig. 6f). Additionally, 5-Aza induced arrest of cells in the G2/M phase under the same conditions as in Fig. 6e, which was significantly reduced upon STAT6 knockdown (Fig. 6g). Collectively, these results suggest that the effects of transient, low-dose 5-Aza on cell death and the cell cycle are primarily, but not exclusively, dependent on STAT6 re-expression in glioma cells.

In addition to its cell-killing effects, transient exposure to low doses of 5-Aza is reported to exert sustained antitumor effects through induction of immune signaling [18, 47]. We further examined whether epigenetic re-expression of STAT6 by 5-Aza is the mechanism mediating expression of immune-related genes. Indeed, several immune-related genes, including interleukin (IL)-1β, C-C motif chemokine ligand (CCL)2, CCL20, IL-8, ICAM1 intercellular adhesion molecule 1 (ICAM1), nuclear factor of activated T cells 2 (NFATC2) and Janus kinase 2 (JAK2), that were significantly induced by low doses of 5-Aza were suppressed upon STAT6 knockdown (Additional file 1: Figure S5), clearly demonstrating dependence of expression on STAT6. Our results support the utility of 5-Aza as an effective chemotherapeutic agent with immune modulating capacity as well as cell-killing effects.

### STAT6 expression is downregulated in glioma stem-like cells

**Fig. 7:**
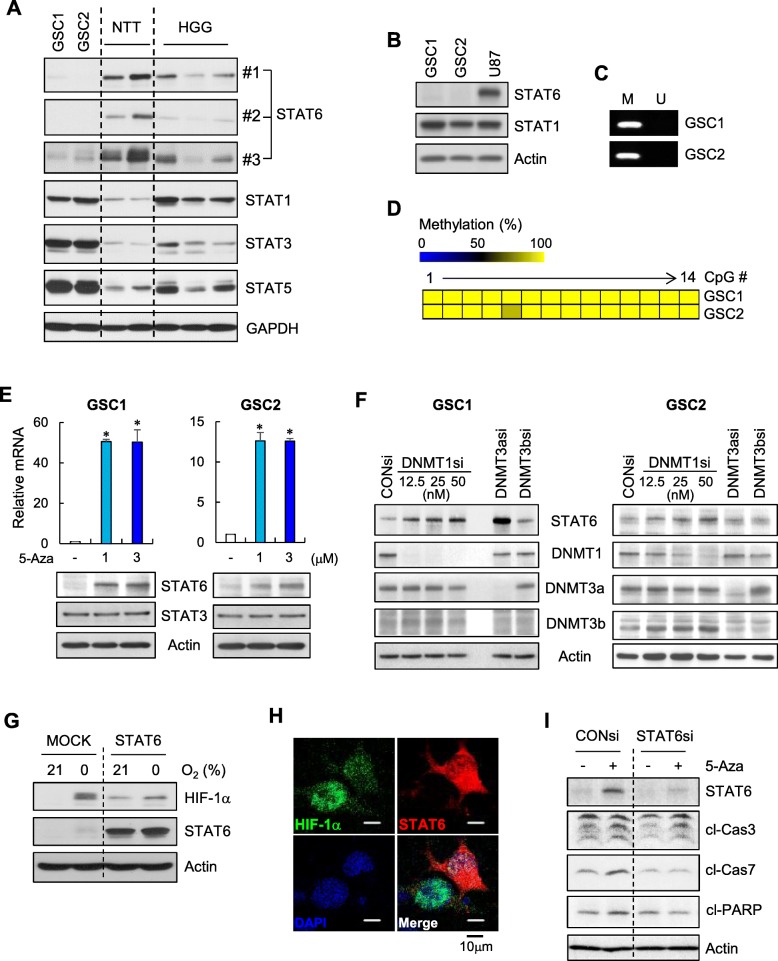
STAT6 expression is downregulated in GSCs. **a** Immunoblot of the indicated STATs in patient-derived GSCs (GSC1 and GSC2), non-tumor brain tissue (NTT), and high-grade glioma (HGG) tissue. STAT6 antibodies: #1, Cell Signaling Technology; #2, BD Biosciences; #3, Santa Cruz. **b-d** Immunoblot of STAT6 and STAT1 (**b**) and MSP (**c**) and bisulfite sequencing (**d**) of *STAT6* promoter CpG islands in GSCs. **e** GSCs were treated with the indicated concentrations of 5-Aza for 72 h, followed by quantification of STAT6 mRNA by RT-qPCR (top) and immunoblotting for STAT6 and STAT3 (bottom). Results are presented as means ± SD (error bars) of three independent experiments (*p < 0.05). **f** Immunoblot of STAT6 and the indicated DNMTs in GSCs transfected with the indicated DNMT siRNA or control siRNA. **g**, **h** Immunoblot of HIF-1α and STAT6 in GSC1 cells transfected with empty vector (MOCK) or Myc-tagged full-length STAT6 (STAT6) and exposed to 21% or 0% O_2_ for 18 h (**g**) and IF staining of HIF-1α in STAT6-transfected GSC1 cells exposed to 0% O_2_ for 18 h (**h**). **i** CONsi- or STAT6si-transfected GSC1 cells were treated as in Fig. 6e, followed by immunoblotting for indicated proteins

Glioma stem-like cells (GSCs) have been implicated in glioma initiation, progression, therapeutic resistance and tumor recurrence, and are thus critical targets for therapy [22]. Therefore, we determined whether our findings could be extended to GSCs. To this end, we established and characterized two patient-derived GSC cell lines, GSC1 and GSC2 (Additional file 1: Figure S6). An examination of STAT protein expression levels showed that STAT6 levels were significantly lower in GSCs compared with non-tumor tissue, whereas levels of STAT1, STAT3 and STAT5 were significantly higher (Fig. 7a). Notably, STAT6 levels in GSCs were even lower than those in high-grade glioma tissues (Fig. 7a). Next, we examined STAT6 promoter methylation status in GSCs. MSP and bisulfite sequencing showed that the *STAT6* promoter was highly methylated in GSCs (Fig. 7b). Furthermore, 5-Aza treatment and DNMT1 knockdown successfully restored STAT6 expression in GSCs (Fig. 7c and d), demonstrating promoter-methylation–dependent STAT6 silencing in GSCs. Intriguingly, although STAT6 silencing was only dependent on DNMT1 in GBM cell lines (Fig. 2), in case of GSC1, both DNMT3a and DNMT1 contributed to STAT6 silencing (Fig. 7d). Finally, we examined the role of STAT6 in HIF-1α accumulation and 5-Aza effects on GSCs. STAT6 overexpression significantly reduced HIF-1α levels in hypoxic GSCs (Fig. 7e and f), indicating that STAT6 downregulation may facilitate GSC survival under hypoxic conditions. In addition, 5-Aza–induced apoptosis were significantly attenuated by STAT6 knockdown.

## Discussion

STATs are dysregulated in a broad range of cancer types. In this study, we demonstrated that STAT6 is downregulated in human glioma specimens as a result of CpG methylation of the *STAT6* promoter. STAT6 downregulation, in turn, promotes HIF-1α protein synthesis under hypoxic conditions through activation of the mTOR signaling pathway, leading to enhanced cell survival. Suppression of mTOR-S6K-S6 by STAT6 is achieved through direct interactions with Rheb. Our experiments indicate that STAT6 restoration using hypomethylating agents, such as 5-azacitidine and decitabine, can promote gliomal cell death in STAT6-silenced GBM. The results obtained in this study extend the potential clinical application of hypomethylating agents for solid tumors, including GBM, requiring multi-modal treatment and precision medicine.

Accumulating evidence indicates that aberrant DNA methylation contributes to GBM pathology [11, 27]. In particular, methylation-specific genomic analyses have identified hypermethylation of notable genes in GBM, including MGMT [49]. Our expression profiling similarly demonstrated frequent downregulation of STAT6 in human GBM specimens as a result of *STAT6* promoter CpG methylation, which is maintained by DNMTs. In our experiments, STAT6 was downregulated under hypoxia in GBM cells, which was reversed by 5-Aza (Additional file 1: Figure S7A and B). Moreover, reoxygenation conditions rescued STAT6 expression (Additional file 1: Figure S7C) and hypoxia-induced promoter methylation was evident with bisulfite sequencing (Additional file 1: Figure S7D). Consistent with our findings, several reports have shown that hypoxia downregulates a number of tumor suppressor genes, including mutL homolog 1 (MLH1), Runt-related transcription factor 3 (RUNX3) and von Hippel–Lindau tumor suppressor (pVHL), through epigenetic mechanisms [19, 23, 29], supporting a potential tumor suppressor function of STAT6.

Hypoxia is a characteristic feature of GBM [16]. Given that HIF-1α is a major regulator in hypoxic environments, our finding that STAT6 negatively regulates HIF-1α suggests an important role in hypoxic GBM. Data from this study showed that STAT6 silencing promotes HIF-1α expression at the translational level via stimulating mTOR-S6K-S6 activity. In keeping with these results, loss of specific tumor suppressors, such as promyelocytic leukemia protein (PML) or tuberous sclerosis proteins 1 and 2 (TSC1 and TSC2), has been shown to promote HIF-1α expression through suppression of hypoxia-induced inhibition of mTOR [2, 4]. Mechanistically, STAT6 suppresses mTOR and downstream S6K and S6 phosphorylation through direct interactions with Rheb. Our data revealed a non-transcriptional function of STAT6, leading to mTOR inhibition. In line with our results, there are several reports regarding non-transcriptional function of STATs. For example, STAT3 is reported to involve in microtubule organization, mitochondrial bioenergetic function, and chromatin regulation [32, 46, 48].

Apart from its inhibitory effects on mTOR signaling, hypoxia promoted transcript and protein expression of 4E-BP1, which was suppressed by STAT6si (Fig. 4d and e). Since 4E-BP1 itself is increased under hypoxia conditions, we were unsure whether the increase in p-4E-BP1 simply reflects an increase in non-functional transcripts but both were suppressed by STAT6si. However, eIF4E phosphorylation and its interactions with eIF4G were significantly enhanced in hypoxic STAT6 knockdown cells (Fig. 4f). Although mTOR is considered a promising target for cancer therapy, targeting mTOR using rapamycin has produced limited beneficial effects in patients [24]. One proposed reason for this unsatisfactory outcome is incomplete targeting of 4E-BP1 phosphorylation [8]. In addition, 4E-BP1 silencing renders protein synthesis resistant to mTOR inhibitors [28]. In this respect, our finding that STAT6 is involved in regulating 4E-BP1 expression could be exploited for rapamycin therapy. Specifically, if 4E-BP1 expression could be regulated by STAT6, STAT6 restoration and the resulting increase in 4E-BP1 could overcome rapamycin-resistant 4E-BP1 phosphorylation in STAT6-silenced GBM. Accordingly, drugs that restore STAT6 expression, such as 5-Aza, may be useful for cancer therapy in combination with rapamycin.

Individual STAT family members not only have discrete functions, they also sometimes play redundant and overlapping roles. For example, constitutively activated STAT3 antagonizes the proapoptotic effects of activated STAT1 in fibroblasts [42]. However, in the absence of STAT1, STAT3 activation by IFN-γ is potentiated and prolonged [38], whereas in MEFs lacking STAT3, IL-6 mediates an IFN-γ-like response, including prolonged activation of STAT1 [9]. Similar to the case of STAT1 and STAT3, it is likely that STAT3 and STAT6 reciprocally regulate each other in GBM. We observed frequent STAT6 downregulation in conjunction with constitutive STAT3 activation in GBM samples tested (Fig. 1a). Further, in malignant glioma cells, it has shown that IL-4 induces aberrant STAT3 activation, but not STAT6 activation [39], suggesting that a defective STAT6 signal might drive STAT3 activation. Although more studies are needed to elucidate the specific relationship between STAT3 and STAT6 in GBM, our data collectively suggest that STAT6 downregulation shifts the balance in favor of GBM pathology.

In addition to its prominent role in adaptive immunity, STAT6 is a critical mediator of viral defense signaling [5]. One significant finding of this study is that STAT6 mediates the induction of chemokines (CCL2, CCL20 and IL-8) by 5-Aza (Additional file 1: Figure S5). Innate immune signaling pathways in host immune and tumor cells have emerged as determinants of responses and resistance to immunotherapy [50]. The activation of tumor-intrinsic innate immune signaling pathways could induce attraction of lymphocytes to tumors, thereby enhancing immunotherapies [31]. Indeed, a recent report has shown that 5-Aza is capable of activating this pathway and inducing sensitization to immune checkpoint blockade [7]. Given that various immunotherapies are being actively studied for treatment of advanced glioma [35], it may be possible that 5-Aza can sensitize GBM cancer cells to immunotherapy, and that STAT6, a critical mediator of chemokine induction, could be a key player in this sensitization mechanism.

GSCs play a central role in cancer initiation and therapeutic resistance, and thus have become a target of GBM therapy [22]. In this context, we demonstrated STAT6 downregulation, its negative regulation of HIF-1α, and its mediation of 5-Aza effects in patient-derived GSCs (Fig. 7). Because HIF-1α is critical for GSC maintenance [37, 43], STAT6 downregulation may favor HIF-1α accumulation in GSCs under hypoxic conditions, thereby contributing to GSC maintenance and GBM progression. Furthermore, 5-Aza is capable of targeting GSCs [40]; thus, restoration of STAT6 may be another mechanism underlying the actions of 5-Aza against GSCs.

Despite recent advances in cancer therapy, the efficacy of anticancer therapeutics remains limited, possibly owing to the complex heterogeneity of cancer cells; thus, some patients still need novel therapeutic agents. The same is true of GBM treatment. Although validation in in vivo, followed by clinical trial is needed, our results suggest that restoration of STAT6 is one such novel therapy. Namely, in GBM in which STAT6 is downregulated, restoring STAT6 would suppress HIF-1α protein synthesis and induce STAT6-regulated immune responses and apoptosis, thereby leading to GMB suppression. Taken together, our data reveal novel roles and mechanisms of action of STAT6 in GBM and propose STAT6 restoration as a promising strategy for GBM therapy.

## Conclusions

Here we show that STAT6 is epigenetically silenced in some cases of malignant glioma, ultimately preventing cancer cell death by inducing HIF-1α expression via the mTOR-S6K-S6 pathway. mTOR activation upon STAT6 knockdown occurs as a result of suppression of direct interactions between STAT6 and Rheb that inhibit HIF-1α translation. Data from our study support a previously uncharacterized role of STAT6 in glioma cell survival and suggest that epigenetic restoration of STAT6 with the aid of DNA methyltransferase inhibitors, including 5-aza-2′-deoxycytidine (decitabine), could be applied as therapy for STAT6-silenced glioma.

## Additional files

Additional file 1: **Figure S1.** Histone acetylation does not contribute to STAT6 silencing. **Figure S2.** STAT6 overexpression suppresses the mTOR-S6K-S6 pathway in U373MG cells during hypoxia. **Figure S3.** STAT6-overexpressing U373MG cells exhibited increased apoptosis during hypoxia. **Figure S4.** Experimental validation for low dose, transient 5-Aza treatment. **Figure S5.** 5-Aza-induced STAT6 expression increases the expression of immune-related genes. **Figure S6.** Characterization of GSCs. **Figure S7.** STAT6 expression is epigenetically silenced during hypoxia. (PDF 990 kb)

Additional file 2: **Table S1.** Detailed information on glioma samples used in this study. **Table S2.** The primers used for qRT-PCR. **Table S3.** Targets CpG islands and the primers for pyrosequencing. **Table S4.** PCR Primers for Expressing Vectors. (PDF 413 kb)

## Data Availability

The datasets used and/or analyzed during the present study are available from the corresponding author on response request.
