# Supplementary material for: Epigenetic downregulation of STAT6 increases HIF-1α expression via mTOR/S6K/S6, leading to enhanced hypoxic viability of glioma cells
Source: Acta Neuropathol Commun. 2019 Sep 17;7:149. doi: 10.1186/s40478-019-0798-z (PMC6747735; doi:10.1186/s40478-019-0798-z)
Supplement: Supplementary file 1 — Figure S1. Histone acetylation does not contribute to STAT6 silencing. Figure S2. STAT6 overexpression suppresses the mTOR-S6K-S6 pathway in U373MG cells during hypoxia. Figure S3. STAT6-overexpressing U373MG cells exhibited increased apoptosis during hypoxia. Figure S4. Experimental validation for low dose, transient 5-Aza treatment. Figure S5. 5-Aza-induced STAT6 expression increases the expression of immune-related genes. Figure S6. Characterization of GSCs. Figure S7. STAT6 expression is epigenetically silenced during hypoxia. (PDF 990 kb) [file 40478_2019_798_MOESM1_ESM.pdf]

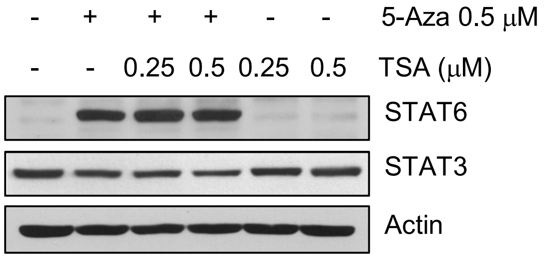

Figure S1. Histone acetylation does not contribute to STAT6 silencing. Related to Figure 2.

Immunoblot of STAT6 in U373 cells treated with or without indicated drugs for 3 days. TSA was treated for the last 1 day.

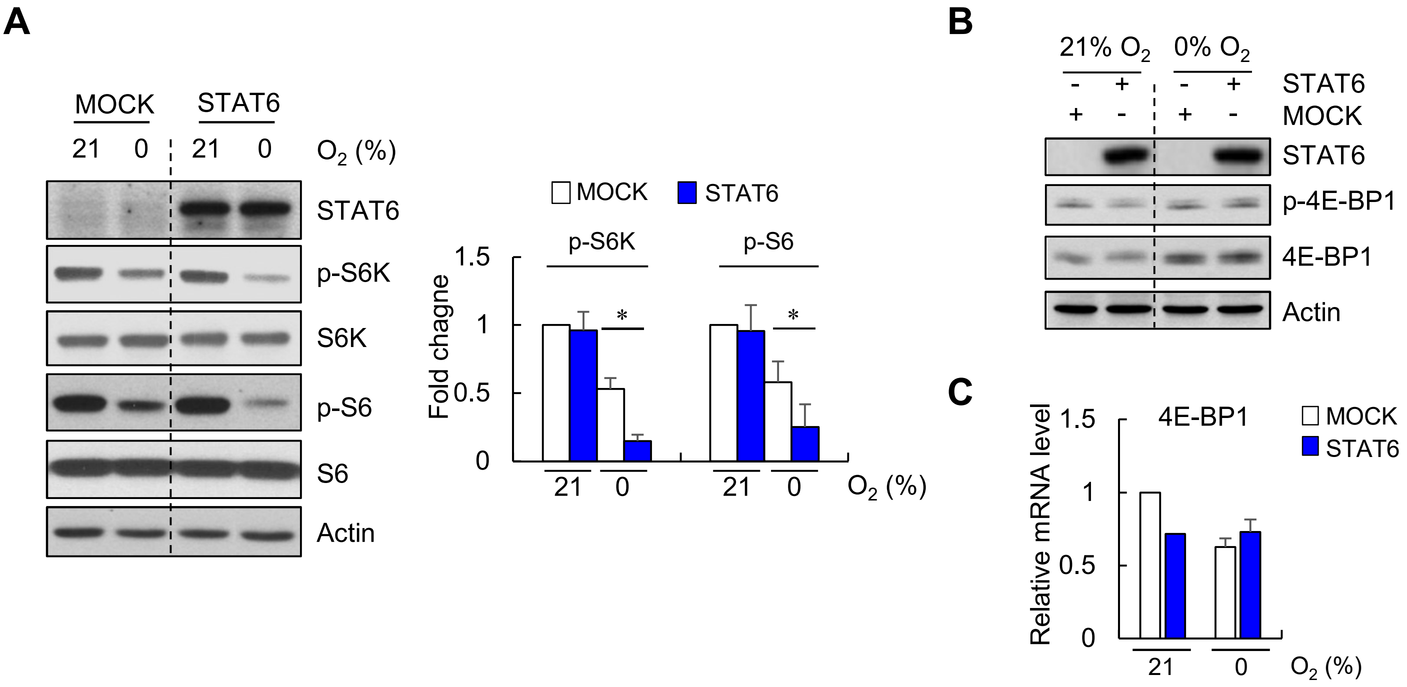

Figure S2. STAT6 overexpression suppresses the mTOR-S6K-S6 pathway in U373MG cells during hypoxia. Related to Figure 4.

A Immunoblot of the indicated proteins (left) and summary data (right) showing actin-normalized p-S6K and p-S6 levels, expressed as fold-changes relative to that in MOCK-transfected cells exposed to 21% O_2 in MOCK- or STAT6-transfected U373MG cells exposed to 0% O_2 for 18 hours. Results are presented as means ± SD (error bars) of three independent experiments (*$p < 0.05$).

B, C MOCK- or STAT6-transfected U373MG cells exposed to 0% O_2 for 18 hours, followed by immunoblotting for 4E-BP (**B**) and quantification of 4E-BP mRNA by RT-qPCR (**C**). Results are presented as means ± SD (error bars) of three independent experiments.

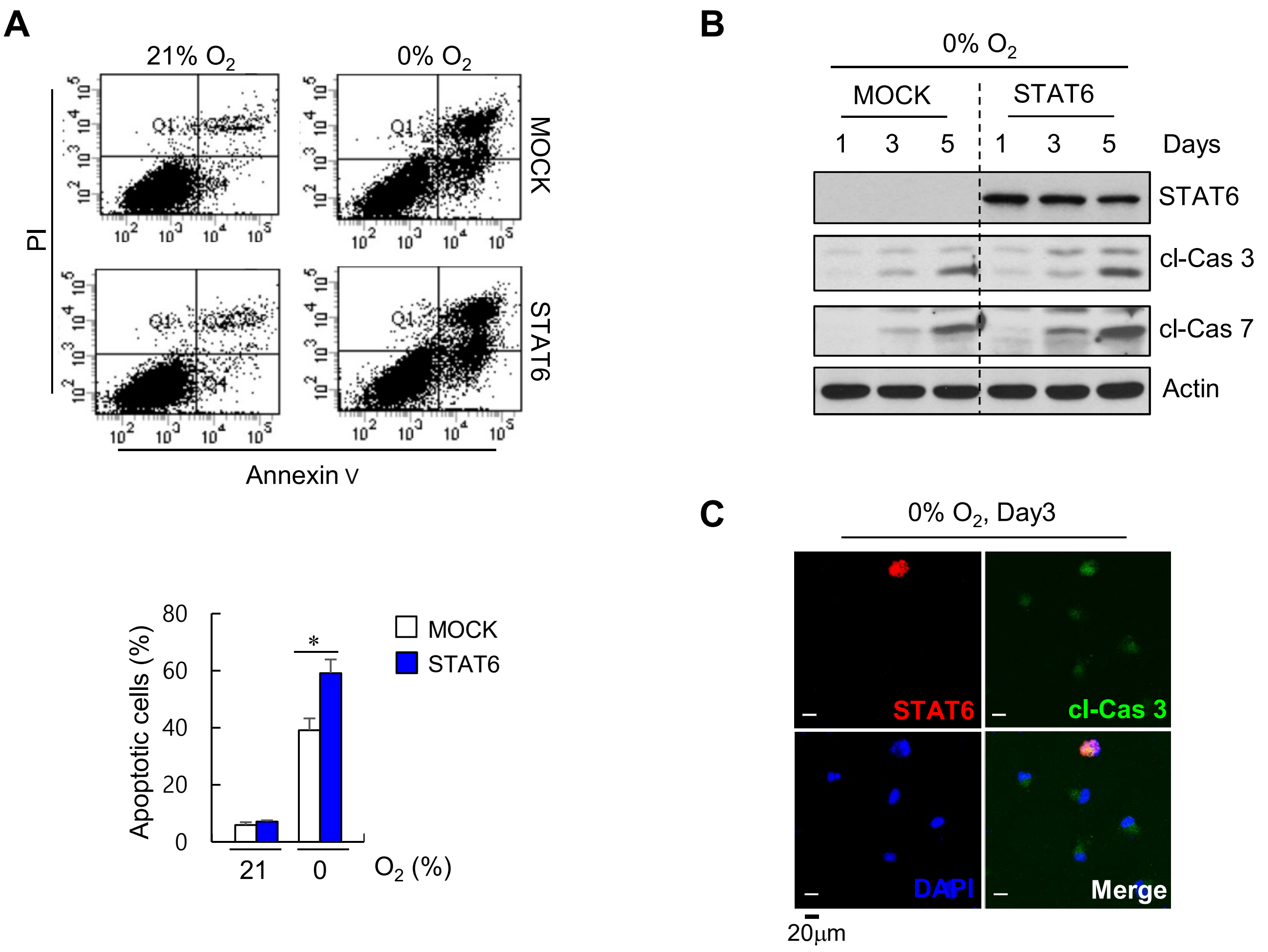

Figure S3. STAT6-overexpressing U373MG cells exhibited increased apoptosis during hypoxia. Related to Figure 6.

A Flow cytometry dot plots of annexin V and propidium iodide (PI) staining and summary data showing the percentage of annexin V-positive cells in MOCK- or STAT6-transfected U373MG cells exposed to 0% O_2 for 5 days. . Results are presented as means ± SD (error bars) of three independent experiments (*$p < 0.05$ vs. MOCK-transfected cells).

B Immunoblot of STAT6, cl-Cas 3, and 7 in MOCK- or STAT6-transfected U373MG cells exposed to 0% O_2 for indicated days.

C Immunofluorescence staining of cl-Cas 3 in STAT6 transfected U373MG cells exposed to 0% O_2 for 3 days.

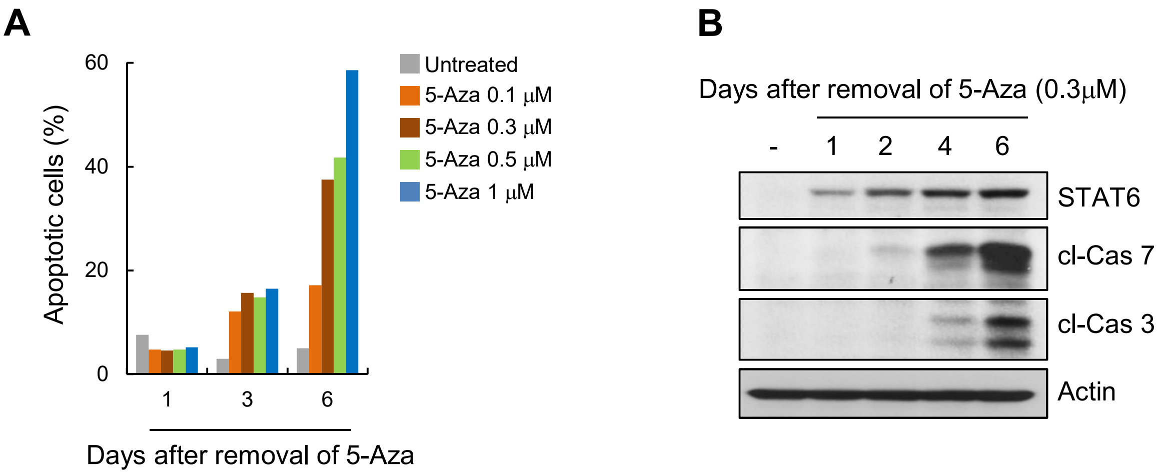

Figure S4. Experimental validation for low dose, transient 5-Aza treatment. Related to Figure 6.

A, B U373MG cells were incubated with or without indicated doses of 5-Aza for 2 days, and then incubated with fresh medium (without drugs) for the indicated days. Summary data showing the percentage of annexin V-positive cells (**A**) and immunoblot of STAT6, cl-Cas 7 and 3 (**B**).

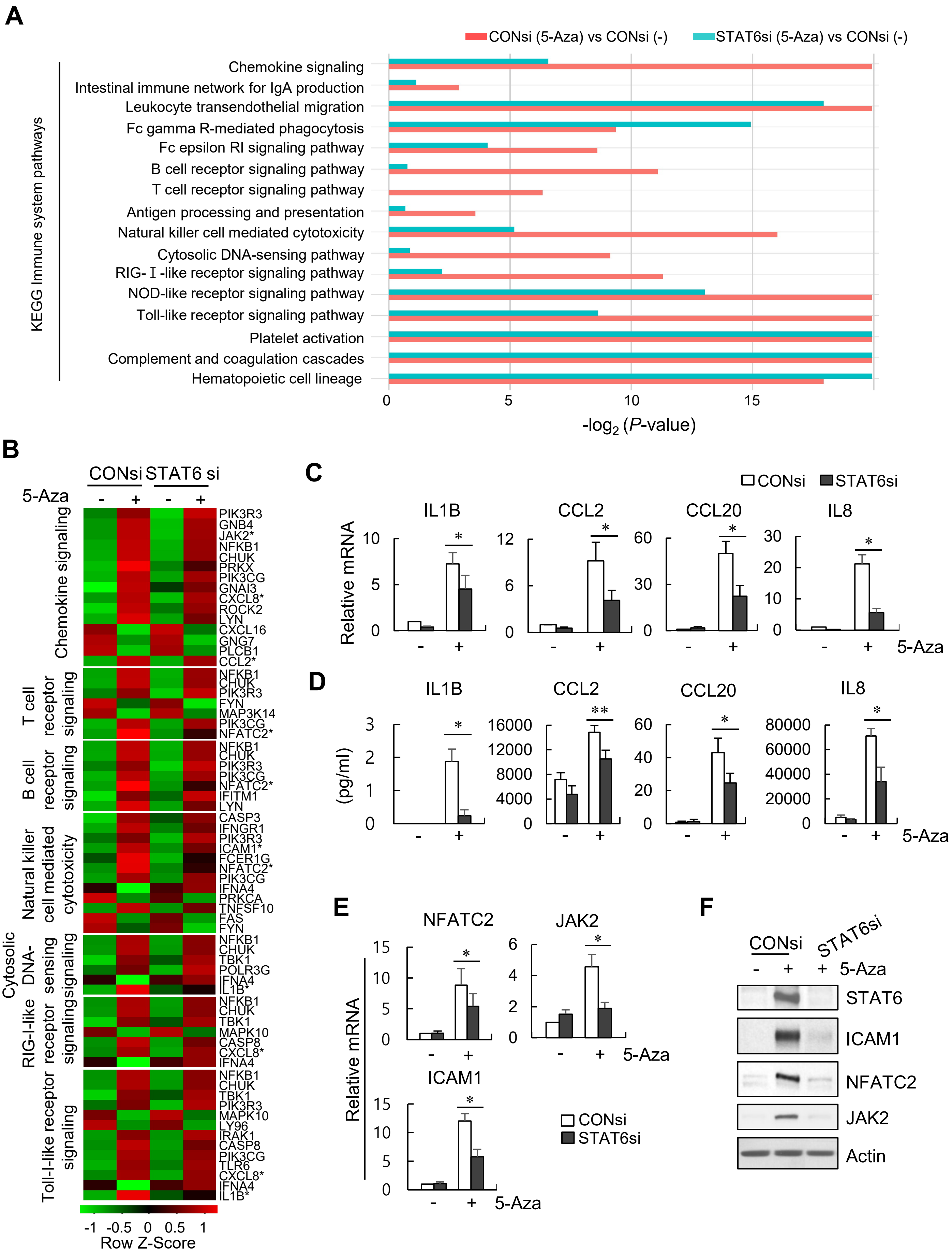

A
CONsi (5-Aza) vs CONsi (-)
STAT6si (5-Aza) vs CONsi (-)
KEGG Immune system pathways
Chemokine signaling
Intestinal immune network for IgA production
Leukocyte transendothelial migration
Fc gamma R-mediated phagocytosis
Fc epsilon RI signaling pathway
B cell receptor signaling pathway
T cell receptor signaling pathway
Antigen processing and presentation
Natural killer cell mediated cytotoxicity
Cytosolic DNA-sensing pathway
RIG-Ⅰ-like receptor signaling pathway
NOD-like receptor signaling pathway
Toll-like receptor signaling pathway
Platelet activation
Complement and coagulation cascades
Hematopoietic cell lineage
0 5 10 15
-log2 (P-value)
B
CONsi STAT6 si
5-Aza - + - +
Chemokine signaling
PIK3R3 GNB4 JAK2* NFKB1 CHUK PRKX PIK3CG GNAI3 CXCL8* ROCK2 LYN CXCL16 GNG7 PLCB1 CCL2*
T cell receptor signaling
NFKB1 CHUK PIK3R3 FYN MAP3K14 PIK3CG NFATC2*
B cell receptor signaling
NFKB1 CHUK PIK3R3 PIK3CG NFATC2* IFITM1 LYN
Natural killer cell mediated cytotoxicity
CASP3 IFNGR1 PIK3R3 ICAM1* FCER1G NFATC2* PIK3CG IFNA4 PRKCA TNFSF10 FAS FYN
Cytosolic DNA-sensing signaling
NFKB1 CHUK TBK1 POLR3G IFNA4 IL1B*
RIG-I-like receptor signaling
NFKB1 CHUK TBK1 MAPK10 CASP8 CXCL8* IFNA4
Toll-I-like receptor signaling
NFKB1 CHUK TBK1 PIK3R3 MAPK10 LY96 IRAK1 CASP8 PIK3CG TLR6 CXCL8* IFNA4 IL1B*
-1 -0.5 0 0.5 1
Row Z-Score
C
CONsi STAT6si
Relative mRNA
IL1B
CCL2
CCL20
IL8
5-Aza
D
(pg/ml)
IL1B
CCL2
CCL20
IL8
5-Aza
E
Relative mRNA
NFATC2
JAK2
ICAM1
5-Aza
CONsi
STAT6si
F
CONsi
STAT6si
- + + 5-Aza
STAT6
ICAM1
NFATC2
JAK2
Actin

Figure S5. 5-Aza-induced STAT6 expression increases the expression of immune-related genes. Related to Figure 6.

A-F CONsi or STAT6si transfected U373MG cells were treated with or without 5-Aza (300nM) for 2 days and rested for 6 days before assaying. Microarray analysis of 5-Aza-induced STAT6 dependent genes. KEGG immune system pathway enrichment analysis for differentially regulated genes (DEGs) in CONsi transfected and STAT6si transfected cells with 5-Aza versus CONsi transfected cells without 5-Aza treatment. Bars indicate statistical significance shown as –log2 of *p* value (**A**). Heat map of DEGs belong to the selected KEGG immune system pathways. Stars indicate genes confirmed by qRT-PCR, ELISA, and/or immunoblot (**B**). qRT-PCR (**C**) and ELISA (**D**) of IL1B, CCL2, CCL20, and CXCL8 and qRT-PCR (**E**) and immunoblot (**F**) of ICAM1, NFATC2, and JAK2 in siRNA transfected U373 cells with or without 5-Aza treatment. Error bars represent the SD of of three independent experiments; $^*p < 0.05$ and $^{**}p < 0.01$.

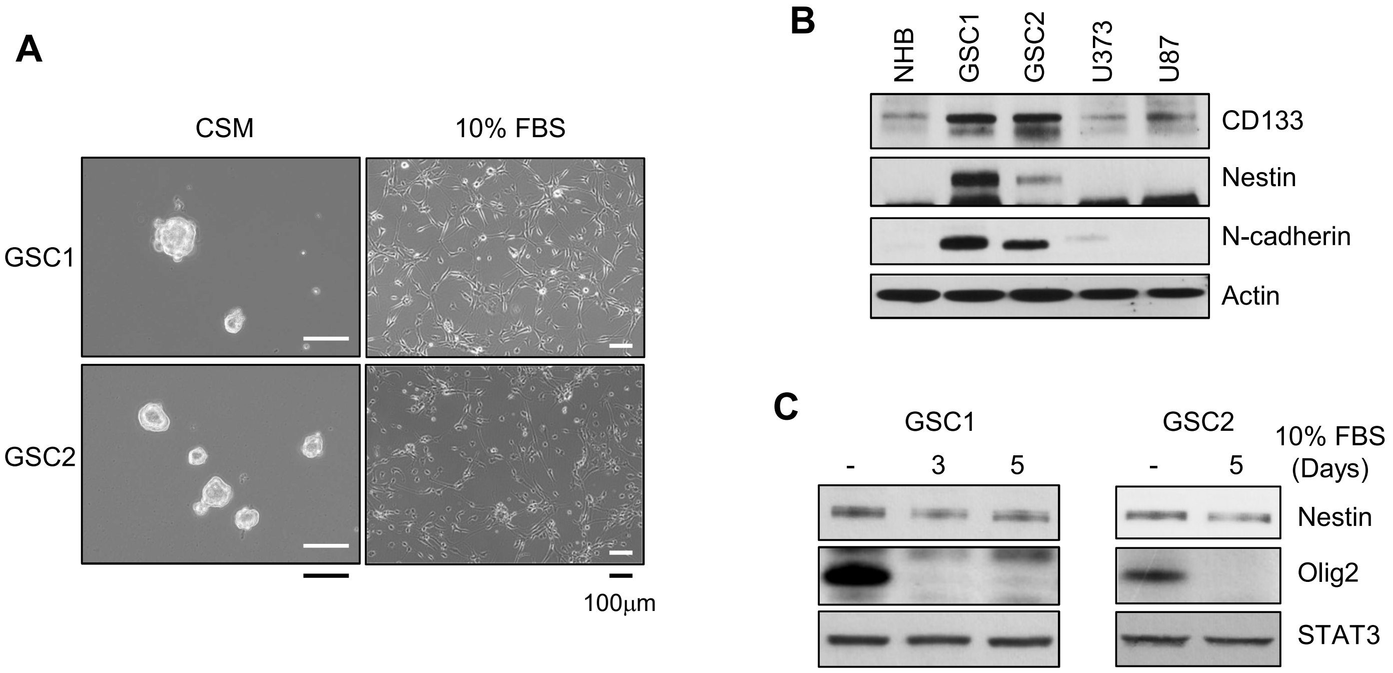

Figure S6. Characterization of GSCs. Related to Figure 7.

A Morphology of GSCs grown as floating spheres in complete stem medium (CSM) and as monolayers after induction of differentiation in serum-containing medium (10% FBS). Scale bars, 100μm.

B Immunoblot of stem cell-related markers (CD133, Nestin and N-cadherin) in the indicated cells.

C Immunoblot of Nestin and Olig2 in GSCs before and after induction of differentiation in serum-containing medium. STAT3 was used as a loading control.

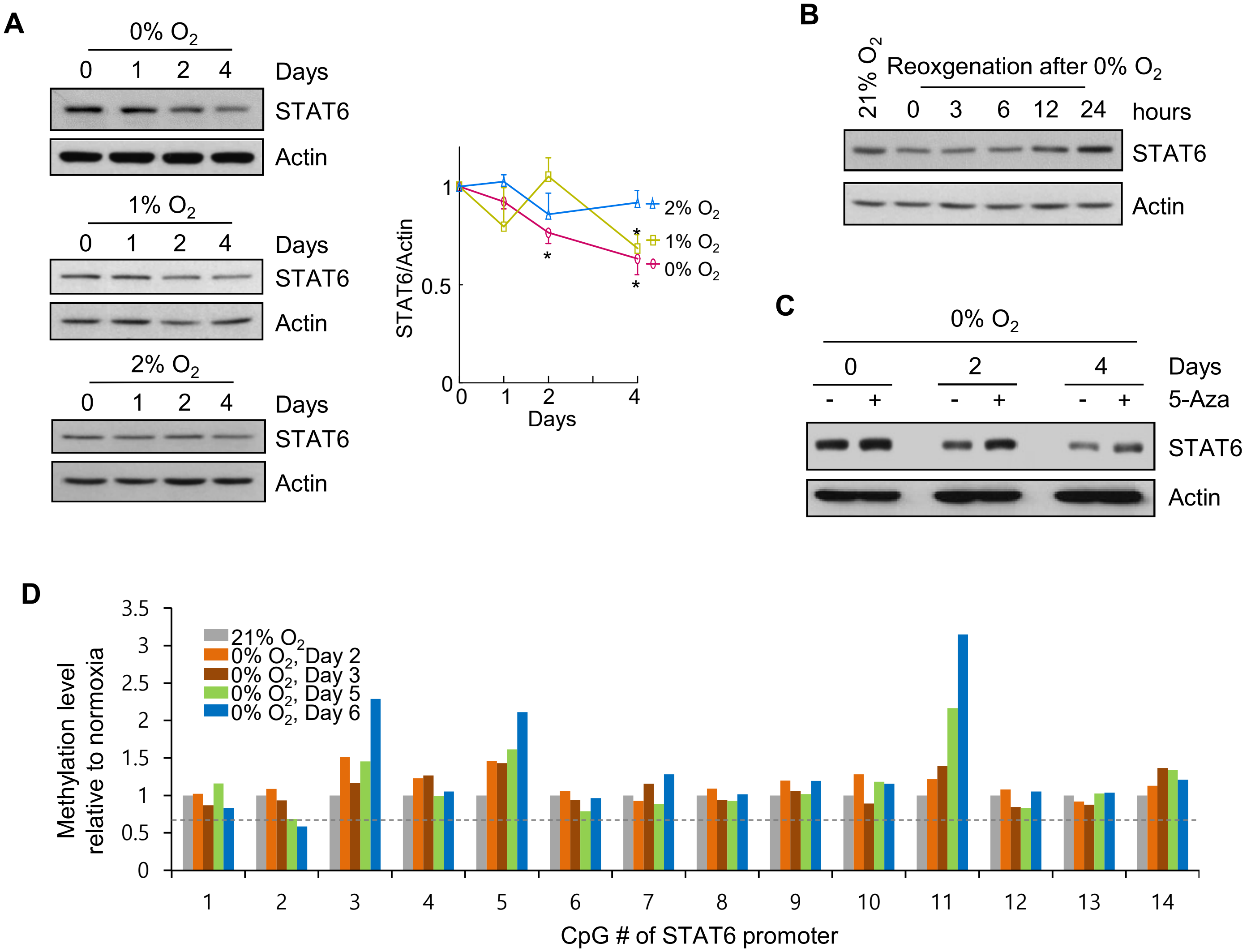

A
0% O2
0 1 2 4 Days
STAT6
Actin
1% O2
0 1 2 4 Days
STAT6
Actin
2% O2
0 1 2 4 Days
STAT6
Actin
STAT6/Actin
1
0.5
0
0 1 2 4
Days
2% O2
1% O2
0% O2
*
B
21% O2
Reoxgenation after 0% O2
0 3 6 12 24 hours
STAT6
Actin
C
0% O2
0 2 4 Days
- + - + - + 5-Aza
STAT6
Actin
D
Methylation level relative to normoxia
3.5
3
2.5
2
1.5
1
0.5
0
21% O2
0% O2, Day 2
0% O2, Day 3
0% O2, Day 5
0% O2, Day 6
1 2 3 4 5 6 7 8 9 10 11 12 13 14
CpG # of STAT6 promoter

Figure S7. STAT6 expression is epigenetically silenced during hypoxia.

A Immunoblot of STAT6 in U87 cells exposed to different hypoxic conditions (0%, 1% or 2% O_2) for the indicated days (top) and summary data (bottom) showing actin-normalized STAT6 levels relative to that in normoxic cells, expressed as fold changes. Results are presented as means ± SD (error bars) of three independent experiments (*$p < 0.05$ vs. normoxic cells).

B Immunoblot of STAT6 in U87 cells exposed to 0% O_2 for 4 days, followed by reoxygenation for the indicated hours.

C Immunoblot of STAT6 in U87 cells exposed to 0% O_2 for the indicated days, with or without 1 μM 5-Aza.

D Bisulfite sequencing of 14 CpG sites in cells exposed to 21% O_2 or the indicated days of 0% O_2. Summary data show the methylation level of individual CpG sites relative to that in 21% O_2.
