# Supplementary material for: Epigenetic downregulation of STAT6 increases HIF-1α expression via mTOR/S6K/S6, leading to enhanced hypoxic viability of glioma cells
Source: Acta Neuropathol Commun. 2019 Sep 17;7:149. doi: 10.1186/s40478-019-0798-z (PMC6747735; doi:10.1186/s40478-019-0798-z)
Supplement: Supplementary file 2 — Table S1. Detailed information on glioma samples used in this study. Table S2. The primers used for qRT-PCR. Table S3. Targets CpG islands and the primers for pyrosequencing. Table S4. PCR Primers for Expressing Vectors. (PDF 413 kb) [file 40478_2019_798_MOESM2_ESM.pdf]

Table S1. Detailed information on glioma samples used in this study

Sample ID	Gender	Age	WHO grade	Diagnosis	IDH1 status	Recurrence
NTT1	M	35	-	Non tumor	-	-
NTT2	M	28	-	Non tumor	-	-
NTT3	M	49	-	Non tumor	-	-
NTT4	M	58	-	Adjacent non tumor HGG17-paired tissue	-	-
NTT5	F	64	-	Adjacent non tumor HGG9-paired tissue	-	-
NTT6	F	73	-	Adjacent non tumor HGG18-paired tissue	-	-
NTT7	F	50	-	Adjacent non tumor HGG14-paired tissue	-	-
NGT1	M	71	-	Metastatic adenocarcinoma	-	-
NGT2	M	62	-	Metastatic adenocarcinoma	-	-
LGG1	M	19	Ⅱ	pleomorphic xanthoastrocytoma	WT	NO
LGG2	F	25	Ⅰ	gangliocytoma	WT	NO
LGG3	F	42	Ⅱ	chordoid glioma	WT	NO
LGG4	M	50	Ⅱ	oligodendroglioma	WT	NO
LGG5	F	36	Ⅱ	ependymoma	WT	NO
LGG6	F	42	Ⅱ	diffuse astrocytoma	R132H	NO
LGG7	F	55	Ⅱ	ependymoma	WT	NO
HGG1	M	72	Ⅳ	glioblastoma	WT	NO
HGG2	M	54	Ⅳ	glioblastoma	WT	NO
HGG3	M	34	Ⅳ	glioblastoma	WT	NO
HGG4	F	64	Ⅳ	glioblastoma	WT	NO
HGG5	F	67	Ⅳ	glioblastoma	WT	NO
HGG6	M	55	Ⅳ	glioblastoma	WT	NO
HGG7	M	73	Ⅳ	glioblastoma	WT	NO
HGG8	F	54	Ⅳ	glioblastoma	WT	NO
HGG9	F	64	Ⅳ	glioblastoma	WT	NO
HGG9-1	F	63	Ⅳ	glioblastoma	WT	YES
HGG10	M	75	Ⅳ	glioblastoma	WT	NO
HGG11	M	40	Ⅲ	anaplastic oligodendroglioma	R132H	NO
HGG12	F	35	Ⅲ	anaplastic oligodendroglioma	R132H	NO
HGG13	F	59	Ⅳ	glioblastoma	WT	NO
HGG14	F	50	Ⅲ	anaplastic oligodendroglioma	R132H	NO
HGG15	F	57	Ⅳ	glioblastoma	WT	NO
HGG16	F	44	Ⅲ	anaplastic oligodendroglioma	WT	YES
HGG17	M	58	Ⅳ	glioblastoma	WT	NO
HGG18	F	73	Ⅳ	glioblastoma	WT	NO

Table S2. The primers used for qRT-PCR

Gene	Forward (5'→3')	Reverse (5'→3')
ICAM1	TAGAGACCCCGTTGCCTAAAA	AGTACACGGTGAGGAAGGTTT
JAK2	TTTGCTGTCGAGCGAGAAAAT	TGAGCGAACAGTTTCCATCTG
NFATC2	ACAGACTGCATCTAACCCCAT	CACAACTTTGGACTCGGATGT
IL1B	ACAGCTGGAGAGTGTAGATCC	TTTTCTGCTTGAGAGGTGCTG
IL8	CTGATTTCTGCAGCTCTGTGT	TGTGGTCCACTCTCAATCACT
CCL2	AGATGCAATCAATGCCCCAG	CAGATCTCCTTGGCCACAATG
STAT6	CAAAGCCCTAGTGCTGAAGAG	CTCCTGCTGTAGCTGGGAATA
HIF1A	CTGACCCTGCACTCAATCAAG	TGGGACTATTAGGCTCAGGTG
PML	TGCAGCTGTATCCAAGAAAGC	TGACTGTACCACAGCCATAGG
BNIP3	CAGCTCACAGTCTGAGGAAGA	GCTTCGGGTGTTTAAAGAGGA
REDD1	TGACCCTGAGGATGAACACTT	CAGTAGTTCTTTGCCCACCTG
CCL20	GCTCCTGGCTGCTTTGATGT	TGCTTGCTGCTTCTGATTCG
4E-BP1	CCATCGTGTGGAGCACTACC	GAGTGAGGGAGAAAGGGCTG
Actin	AGAGCTACGAGCTGCCTGAC	AGCACTGTGTTGGCGTACAG

Table S3. Targets CpG islands and the primers for pyrosequencing

Gene	Primer (5'→3')		Size(bp)
Stat6 (*+807)*	Forward	GTGGTTTAGAAGAGGGGGAATTTT	174
	Biotinylated-reverse	AACAACTTCTCTAAATTACCCAATAC	
	Sequencing (+807)	TGTTGTAGAAGTTGAGATTT	
	Sequencing (+854)	GTGTTGAATTGGTTTTATTTT	
Stat6 (*+967)*	Forward	ATTGGGTAATTTAGAGAAGTTGTTTTAA	164
	Biotinylated-reverse	AACCAATATTACCTCCCAAACT	
	Sequencing (+913)	ATTTAGAGAAGTTGTTTTAAT	
	Sequencing (+967)	AGATAGGGGTTGAGATA	

Table S4. PCR Primers for Expressing Vectors

	Forward (5'→3')	Reverse (5'→3')
STAT6-d1	CTGGATGAAGTCCTGAGAACCCT CGTCAC'	GGCGATCGCGGCGGCAGAT CTCCTC
hSTAT6-d2	GCCTTCTCTGAGATGGACCGCGTGC	GAGGGTTCTCAGGACTTCATCC AGCCG
hSTAT6-d3	GTCCTGGACCTCACCAAACGCTGTC	ATTGTCCCACAGGATAGTGGCT TTGG
hSTAT6-d4	ATCCCAAGAAGCCCAAGGATGAGGC	CCGGAGACAGCGTTTGGTGAG GTCC
